# Tutton salt (NH_4_)_2_Zn(SO_4_)_2_(H_2_O)_6_: thermostructural, spectroscopic, Hirshfeld surface, and DFT investigations

**DOI:** 10.1007/s00894-024-06089-7

**Published:** 2024-09-17

**Authors:** João G. de Oliveira Neto, Jailton R. Viana, Kamila R. Abreu, Luiz F. L. da Silva, Mateus R. Lage, Stanislav R. Stoyanov, Francisco F. de Sousa, Rossano Lang, Adenilson O. dos Santos

**Affiliations:** 1 Center for Social Sciences, Health, and Technology, Federal University of Maranhão-UFMA, Imperatriz, MA 65900-410 Brazil; 2 Institute of Criminalistics, Scientific Police of Pará, Marabá, PA 68507-000 Brazil; 3 CanmetENERGY Devon, Natural Resources Canada, 1 Oil Patch Drive, Devon, AB T9G 1A8 Canada; 4 Institute of Exact and Natural Sciences, Federal University of Pará–UFPA, Belem, PA 66075-110 Brazil; 5 Institute of Science and Technology, Federal University of São Paulo-UNIFESP, São José Dos Campos, SP 12231-280 Brazil

**Keywords:** Tutton salts, (NH_4_)_2_Zn(SO_4_)_2_(H_2_O)_6_, Hirshfeld surfaces, DFT calculations, Thermochemical devices, Heat storage materials

## Abstract

**Context:**

Ammonium Tutton salts have been widely studied in recent years due to their thermostructural properties, which make them promising compounds for application in thermochemical energy storage devices. In this work, a detailed experimental study of the Tutton salt with the formula (NH_4_)_2_Zn(SO_4_)_2_(H_2_O)_6_ is carried out. Its structural, vibrational, and thermal properties are analyzed and discussed. Powder X-ray diffraction (PXRD) studies confirm that the compound crystallizes in a structure of a Tutton salt, with monoclinic symmetry and *P*2_1_/*a* space group. The Hirshfeld surface analysis results indicate that the main contacts stabilizing the material crystal lattice are H···O/O···H, H···H, and O···O. In addition, a typical behavior of an insulating material is confirmed based on the electronic bandgap calculated from the band structure and experimental absorption coefficient. The Raman and infrared spectra calculated using DFT are in a good agreement with the respective experimental spectroscopic results. Thermal analysis in the range from 300 to 773 K reveals one exothermic and several endothermic events that are investigated using PXRD measurements as a function of temperature. With increasing temperature, two new structural phases are identified, one of which is resolved using the Le Bail method. Our findings suggest that the salt (NH_4_)_2_Zn(SO_4_)_2_(H_2_O)_6_ is a promising thermochemical material suitable for the development of heat storage systems, due to its low dehydration temperature (≈ 330 K), high enthalpy of dehydration (122.43 kJ/mol of H_2_O), and hydration after 24 h.

**Methods:**

Computational studies using Hirshfeld surfaces and void analysis are conducted to identify and quantify the intermolecular contacts occurring in the crystal structure. Furthermore, geometry optimization calculations are performed based on density functional theory (DFT) using the PBE functional and norm-conserving pseudopotentials implemented in the Cambridge Serial Total Energy Package (CASTEP). The primitive unit cell optimization was conducted using the Broyden–Fletcher–Goldfarb–Shanno (BFGS) algorithm. The electronic properties of band structure and density of states, and vibrational modes of the optimized crystal lattice are calculated and analyzed.

**Graphical abstract:**

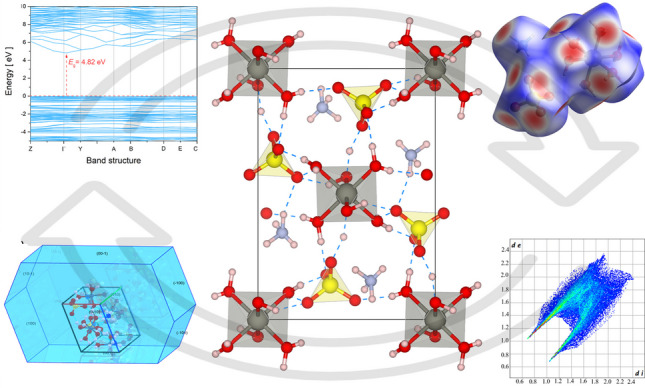

**Supplementary Information:**

The online version contains supplementary material available at 10.1007/s00894-024-06089-7.

## Introduction

Double salt hydrates with a picromerite-type structure have attracted attention due to their promising melting temperatures, high enthalpies of fusion, energy storage densities, and diverse thermostructural properties, offering a wide range of applications in the area of thermal energy storage [1, 2]. For instance, Tutton salts have been widely explored in recent decades as compounds for thermochemical energy storage [3, 4].

Tutton salts form crystals with the chemical formula M_2_M*(XO_4_)_2_(H_2_O)_6_, where M is a monovalent cation (Na^+^, K^+^, NH_4_^+^, Rb^+^ or Cs^+^), M* is a divalent cation of alkaline earth metal, such as Mg^2+^, and 3*d* block transition metals, for example, Mn^2+^, Fe^2+^, Co^2+^, Ni^2+^, Cu^2+^, and Zn^2+^, and X can be occupied by a chalcogenic element, typically, S or Se [5, 6]. These crystals present monoclinic symmetry with a space group of *P*2_1_/*a*, two formulas per unit cell (*Z* = 2), and three distinct moieties M, [M*(H_2_O)_6_], and (XO_4_) co-crystallized in the structural lattice. The [M*(H_2_O)_6_] moiety, in which the H_2_O molecules form a slightly distorted octahedron around the divalent metal ion, is particularly important due to the occurrence of the Jahn–Teller effect [7, 8]. This physical phenomenon is observed in several transition metal complexes with a square bipyramidal geometry, in which the *d*-level atomic orbitals are split into two degenerate states [9]. If the molecular unit has a degenerate ground state with octahedral symmetry, a lower energy state is formed because of a Jahn–Teller distortion that removes the degeneracy and lowers the symmetry [10].

Based on the literature, several works report the synthesis, structure determination, and nuclear magnetic resonance spectra of the zinc(II) Tutton salt crystals, such as Na_2_Zn(SO_4_)_2_(H_2_O)_6_ [11], K_2_Zn(SO_4_)_2_(H_2_O)_6_ [12], Rb_2_Zn(SO_4_)_2_(H_2_O)_6_ [11], Cs_2_Zn(SO_4_)_2_(H_2_O)_6_ [13], and (NH_4_)_2_Zn(SO_4_)_2_(H_2_O)_6_ [14]. Under ambient conditions, all of these Zn(II) double salts exhibit similar lattice parameters and molecular structure, as they belong to an isomorphic class [15]. Additionally, in these five different compositions, the zinc dications occupy the (0,0,0) and (1/2,1/2,0) positions within the unit cell in a symmetrical inversion center (*C*_i_), while the other atoms and molecules assume general positions [16]. Limited research has been conducted to investigate experimentally the thermostructural properties on these materials, as well as to gain computational insights into the effect of the chemical composition of each inorganic salt on the crystal structure and properties [17].

Density functional theory (DFT) has become well established as a reliable and effective computational chemistry tool for the accurate calculation of valuable structural, vibrational, and electronic properties of single molecules, aggregates, and crystalline systems. The DFT method has been employed to support experimental data and explore the crystal structure and thermodynamic properties of constituent moieties in Tutton salts [18].

Hirshfeld surface analysis is a computational technique widely used to study the intermolecular contacts among molecular units in crystals [19]. This technique enables the qualitative and quantitative evaluation of non-covalent interactions among chemical species within a crystal lattice. Qualitatively, it generates three-dimensional (3D) surfaces to analyze the electron density distribution over adjacent molecules, based on interatomic distances. The quantitative analysis produces general and specific histograms, allowing us to identify and measure the contribution of each intermolecular contact in the crystal structure. The Hirshfeld surface analysis is based on the partitioning of the crystal into regions, in which the sum of the electronic density of the promolecule dominates the sum over the procrystal. The resultant isosurface provides information about intermolecular interactions [20, 21].

Another very useful theoretical method for understanding the structure of double salts is the crystal voids analysis. In this method, procrystal electron density isosurfaces are projected onto the unit cell of a system to locate, visualize, and quantify the free space in the crystalline materials. The void surfaces are calculated from the sum of spherical atomic electron densities, which are positioned at the appropriate nuclear coordinates obtained from experimental X-ray diffraction (XRD) data [22].

Despite the limitations of some experimental and theoretical studies, several reports already include the combined experimental-computational studies of Tutton salts. Particular examples include (NH_4_)_2_ M*(SO_4_)_2_(H_2_O)_6_ with M* = Mn, Ni, or Cu [23, 24], (NH_4_)_2_Mn_1-x_Zn_x_(SO_4_)_2_(H_2_O)_6_ [4], and K_2_Zn(SO_4_)_2_(H_2_O)_6_ [25].

Several researchers have focused their effort on studying the Tutton salt (NH_4_)_2_Zn(SO_4_)_2_(H_2_O)_6_. This crystal was first synthesized in 1964 by Montgomery and Lingafelter, who elucidated its structure using single-crystal XRD [26]. Posteriorly, Barashkov et al. [27] carried out a comparative study of Raman and infrared (IR) spectroscopy of the Tutton salts K_2_Zn(SO_4_)_2_(H_2_O)_6_ and (NH_4_)_2_Zn(SO_4_)_2_(H_2_O)_6_ based on the isolated fragments inherent to each composition. Additionally, electronic paramagnetic resonance studies as a function of temperature (4.2–300 K) were conducted by Hoffmann and Radczyk [14] to verify the influence of NH_4_^+^ tetrahedra on the structure. Furthermore, structural searches involving mixed Tutton salts with compositions (NH_4_)_2_Mn_1-x_Zn_x_(SO_4_)_2_(H_2_O)_6_ and (NH_4_)_2_Cr_1-x_Zn_x_(SO_4_)_2_(H_2_O)_6_ were conducted by Oliveira Neto et al. [4] and Cotton et al. [28], respectively. However, despite the aforementioned research, to date, phase change temperatures, determination of new structural phases at high temperature, thermal expansion coefficients, and vibrational properties have not been explored in relation to intermolecular interactions.

In this work, we report the growth of (NH_4_)_2_Zn(SO_4_)_2_(H_2_O)_6_ crystal, here named as NHZnSO, and characterize and discuss its structural, spectroscopic, and thermal properties. The main objective of this article was to conduct a thorough analysis of intermolecular interactions and IR and Raman vibrational modes using Hirshfeld surfaces and DFT-based first-principles calculations of the crystal lattice. In addition, we performed a simultaneous thermostructural study to elucidate the influence of temperature on NHZnSO crystals, aiming to identify and determine new structural phases with potential applications in thermochemical energy storage. This research aimed to provide insights based on a combined experimental-theoretical approach, shedding light on the key intermolecular interactions in this class of materials.

## Experimental and theoretical methods

### Crystal growth

**Fig. 1:**
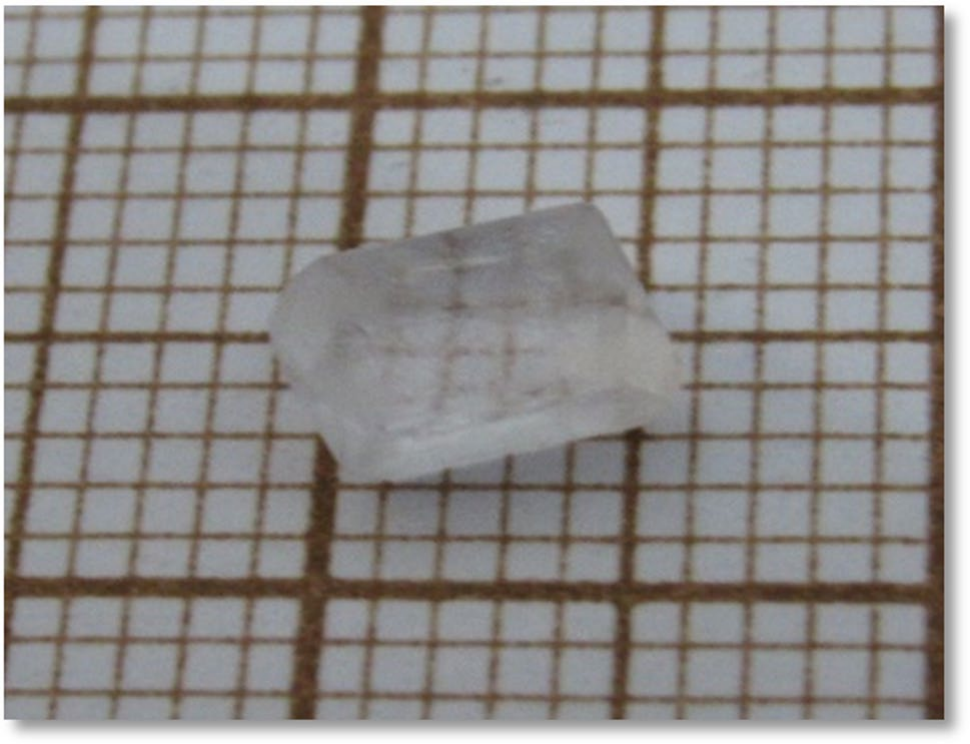
Photograph of as-grown NHZnSO crystal by the solvent slow evaporation technique

The NHZnSO crystals were synthesized from a saturated solution using the slow solvent evaporation method at 308 K. For that, the reagents (NH_4_)_2_SO_4_ and ZnSO_4_(H_2_O)_7_ (both from the company Sigma–Aldrich, > 99%) were used as starting compounds in a stoichiometric ratio of 1:1 (0.6607 g: 1.4377 g, respectively). Firstly, these materials were homogenized simultaneously in deionized water (50 mL) under magnetic stirring for 5 h. After the complete dissolution of the compounds, the final solution with a pH of 4.33 was filtered and then allowed in an oven at 308 K to undergo slow solvent evaporation and nucleation of the solid phase. After 3 weeks, colorless single crystals with average dimensions of around 0.70 × 1.22 × 0.25 cm^3^ (L × W × H) were successfully obtained, as presented in Fig. 1.

### Material characterizations

The structure of the NHZnSO crystal was analyzed using powder X-ray diffraction (PXRD) at room conditions and high temperatures using a PANalytical diffractometer (Empyrean) with Cu-Kα radiation (λ = 1.5418 Å), operating at 40 kV/40 mA. The diffractograms were recorded from 10 to 40° (2θ) angular range with a step size of 0.02° and a counting time of 2 s/step. For the measurements as a function of high temperature, the same instrumental procedures were employed in the temperature range between 298 and 623 K, from an Anton-Paar TTK450 temperature chamber. Furthermore, the crystalline phase was identified by the Rietveld refinement method [29] from the EXPGUI-GSAS software [30] based on crystallographic parameters previously related in the literature [31].

Transmittance, absorbance, and reflectance spectra were measured in the 200–800 nm region. These spectra were acquired using an Evolution 220 UV–Vis–NIR spectrophotometer with dual-beam and a deuterium lamp from Thermo Scientific.

Fourier-transform infrared (FT-IR) spectrum was collected from the crystal powder using a PerkinElmer spectrometer containing an attenuated total reflectance accessory (UATR) consisting of a diamond/ZnSe crystal (1 reflection). The data were measured in the wavenumber range from 4000 to 400 cm^−1^, with a spectral resolution of 4 cm^−1^.

The Raman spectrum at room conditions was recorded using a LabRAM Raman spectrometer (HR Evolution Horiba) in the 50–3600 cm^−1^ spectral region. The sample was excited by the 633 nm line of a red solid-state laser with 2 mW of average laser power on the sample surface. The spectrum was collected with 4 accumulations of 120 s time each, and a spectral resolution of 4 cm^−1^.

Thermoanalytical measurements were conducted using thermogravimetry (TG) and differential thermal analysis (DTA) simultaneously from a Shimadzu DTG-60 thermal analyzer. The TG–DTA thermograms were measured in the temperature interval between 303 and 723 K, under a nitrogen atmosphere with a flow rate of 100 mL/min and a heating rate of 10 K/min. These parameters were applied to a fine powder sample weighing around ≈ 4.97 mg distributed evenly on an alumina crucible. Additionally, differential scanning calorimetry (DSC) was performed on a Shimadzu DSC-60 thermal analyzer. The measurement was carried out in the 303 and 723 K temperature range with a heat rate of 10 K/min and a dry N_2_ gas flow rate of 100 mL/min. A powder sample of ≈ 2.23 mg was deposited in an aluminum crucible to start the thermal experiment.

### Hirshfeld surfaces crystal voids

The 3D Hirshfeld surfaces and two-dimensional (2D) fingerprint graphs were plotted using the Crystal Explorer 17 software [32] to identify and quantify intermolecular contacts occurring in the primitive unit cells. These data were generated from the crystallographic information file (.cif) 65013, which contains the previously solved structure of NHZnSO crystal and is available in the Inorganic Crystal Structure Database (ICSD). The 3D maps and their analogs were designed with the normalized distance property (*d*_norm_), identified in terms of the distances from a given point on the surface to the nearest external (*d*_e_) and internal (*d*_i_) atom and van der Waals radius (*r*_vdW_). Additionally, 2D fingerprint graphs were plotted as a function of *d*_e_ and *d*_i_, covering all intermolecular interactions to quantify specific contacts in terms of the percentage of each contribution [33].

The crystal voids analysis methodology was applied to indicate the free spaces in the crystal structure [22]. The voids were generated through crystal electronic density isosurfaces of 0.002 a.u., as proposed by Bader et al. [34].

### DFT calculations

The DFT implementation in the Cambridge Serial Total Energy Package (CASTEP) [35] was employed to conduct calculations on the structural, geometric, electronic, and vibrational properties of the NHZnSO crystal structure. CASTEP is a first-principles quantum mechanics-based program, utilizing plane wave basis sets and pseudopotentials for materials modeling. Similar to the computational study using Hirshfeld surfaces, the initial structure of the NHZnSO crystal was taken from the crystallographic information file under code 65013 obtained from the ICSD crystallographic database and used as input in the DFT calculations. The generalized gradient approximation (GGA) with the Perdew, Burke, and Ernzerhof (PBE) functional [36] was employed in the calculations. The GGA-PBE functional was selected as it has been widely used in the literature and is known to provide significant accuracy for geometric and spectroscopic parameters, particularly for hydrated inorganic systems [25, 37]. This makes it widely used for computational studies that aim to achieve a balanced comparison between calculated and experimental data. Norm-conserving pseudopotentials (Supplementary Material) were used to account for electron–ion interactions outside the core region, ensuring an accurate description of their scattering properties [38]. A Monkhorst–Pack grid with a medium 3 × 2 × 2 K-set point [39] was employed, and an energy cut-off of 700 eV with a selected energy tolerance per atom was considered. These grid parameters were selected based on DFT calculations performed on similar materials previously reported in the literature [37, 40]. The primitive unit cell optimization was conducted using the Broyden–Fletcher–Goldfarb–Shanno (BFGS) algorithm [40]. For the geometry optimization convergence, the maximum energy change was set to 1.0 × 10^−5^ eV/atom, the maximum force to 0.05 eV/Å, the maximum stress to 0.1 GPa, and the displacement to 0.001 Å. The electronic wave function propagated in the reciprocal lattice through a high symmetry path in the Brillouin zone of the cubic crystal, along the points Z (0.000, 0.000, 0.500); G(Γ) (0.000, 0.000, 0.000); Y (0.000, 0.500, 0.000); A (− 0.500, 0.500, 0.000); B (− 0.500, 0.000, 0.000); D (− 0.500, 0.000, 0.500); E (− 0.500, 0.500, 0.500); and C (0.000, 0.500, 0.000). For this calculation, a total of 78 ions in a monoclinic phase (*P*2_1_/*a*-space group) were utilized.

Vibrational frequencies were determined by calculating the spatial derivatives of macroscopic polarizations, following the methodology outlined by Porezag and Pederson [42]. The numerical computation of spatial derivatives of macroscopic polarization was conducted along the eigenvectors associated with each Raman active phonon mode. The polarization for each displacement was acquired using the linear response formalism. The Raman susceptibility tensor, crucial for Raman intensity calculations, is defined according to Eq. (1):

1 $${A}_{\alpha \beta }^{m}=\sqrt{V}\sum\limits_{I\gamma }\frac{d{\chi }_{\alpha \beta }^{\left(1\right)}}{d{R}_{I\gamma }}\frac{{\upsilon }_{I\gamma }^{m}}{\sqrt{{M}_{I}}}$$

where ${\chi }_{\alpha \beta }^{\left(1\right)}$ is the first order dielectric susceptibility, and *υ* is the phonon eigenvector in the direction in which the atom *I*, at equilibrium positions *R*, moves under excitation of a phonon mode *m* in a unit cell with volume (*V*). The calculation was evaluated using norm-conserving pseudopotential [38].

## Results and discussion

### PXRD and Rietveld refinement

**Fig. 2:**
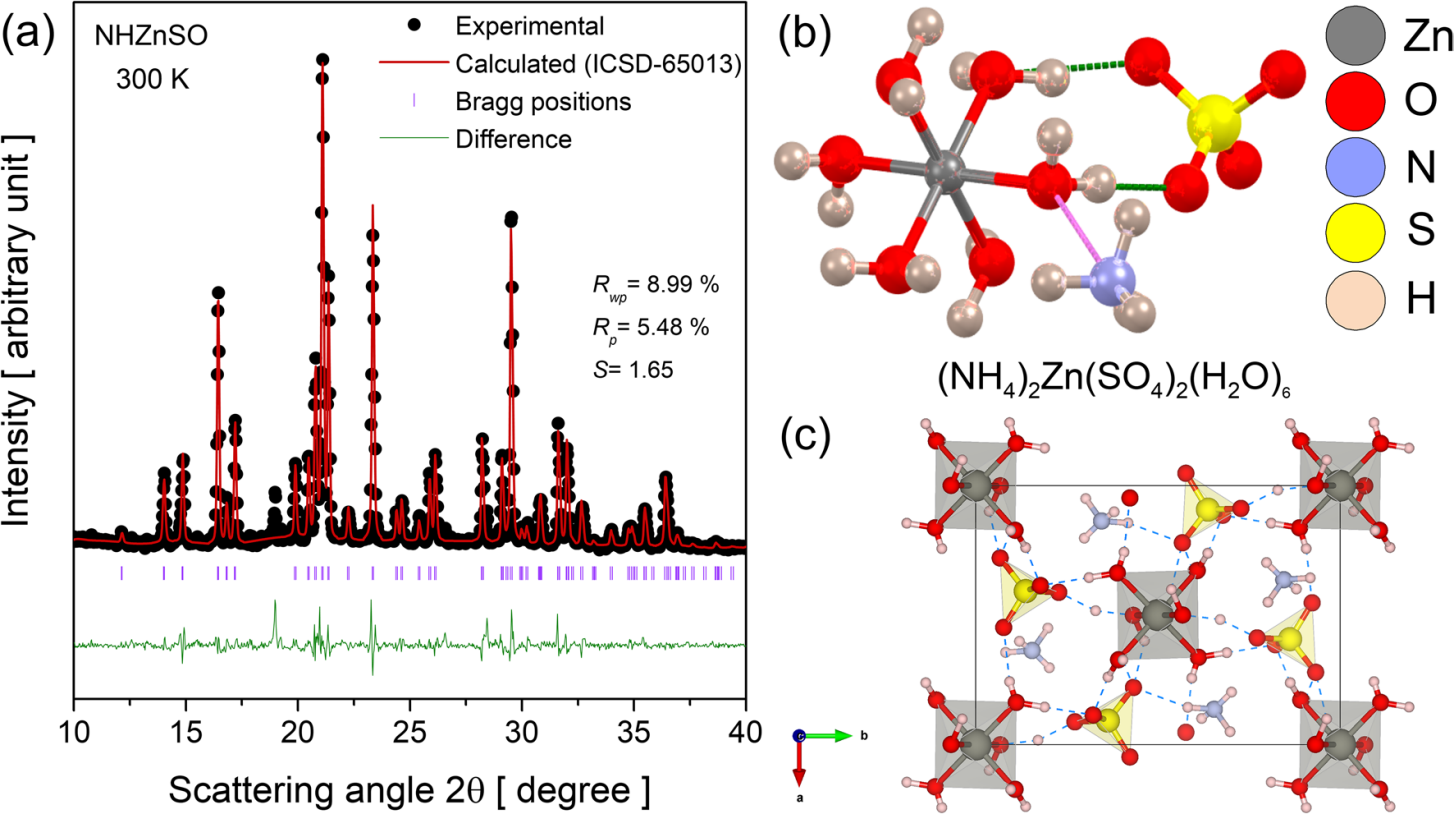
**a** PXRD pattern of NHZnSO refined by the Rietveld method at 300 K; **b** relaxed structure; **c** unit cell with a monoclinic symmetry along the *c*-axis showing the lattice of hydrogen bonds formed by the H_2_O molecules with ammonium and sulfate moieties

The experimental PXRD pattern (gray open circles) of NHZnSO crystal at room conditions (300 K) refined using the Rietveld refinement method (red line) with the Bragg positions (orange lines) and the difference between the intensities (green line) are presented in Fig. 2a. The Rietveld method was applied to the PXRD pattern to identify the crystalline phase and determine the structural parameters based on a comparative analysis between experimental and theoretical (ICSD-65013) data. Employing a least squares approach, the Rietveld method refined the theoretical line profile until it aligned with the measured pattern [29]. This analysis revealed that the sample crystallizes in a monoclinic system of *P*2_1_/*a* (n° 14) (${C}_{2h}^{5}$) space group with two formulas of [(NH_4_)_2_Zn(SO_4_)_2_(H_2_O)_6_] per unit cell, as expected for Tutton salts. The lattice parameters obtained are *a* = 9.343(4) Å, *b* = 12.648(8) Å, *c* = 6.241(5) Å, *α* = γ = 90.00°, *β* = 109.92(3)°, and *V* = 705.65(9) Å^3^. These values indicate that there is a good correlation between literature data (ICSD-65013) [30] and the experimental PXRD pattern obtained here. The peak at 2θ = 19.02° indicates the presence of small amounts of ZnSO_4_⋅7H_2_O. Table S1 provides the structural parameters previously reported and the experimental data refined using the Rietveld method, in which it can be observed that the difference between the crystallographic parameters is less than 3.5%. Moreover, the factors *R*_wp_ (weighted residual), *R*_p_ (least squares refinement residual), and *S* (goodness of fit), which presented values of 8.99%, 5.48%, and 1.65, respectively, confirm the good agreement between the results shown here and ICSD-65013 [30]. It is worth highlighting that the main reflections $(110)$; ($0 2 0$); ($0 0 1$); ($0 1 1$); ($1 1\overline{ 1 }$); ($1 2 0$); ($2 0 0$); ($0 2 1$); ($1 2 \overline{1 }$); ($2 1 0$); ($1 3 0$); ($1 3\overline{ 1 }$); ($3 0\overline{ 1 }$); ($2 2 1$); ($12\overline{2 }$); and ($3 1\overline{ 2 }$) are respectively positioned at 2θ =  12.2°; 14.0°; 14.7°; 16.4°; 16.8°; 17.3°; 20.0°; 20.4°; 20.8°; 21.2°; 23.4°; 26.3°; 29.4°; 31.7°; 32.1°; and 36.6°.

Figure 2b illustrates the optimized structure of NHZnSO crystal with a lattice of hydrogen bonds among water, ammonium, and sulfate moieties (dashed green and pink lines). As noted, the structure of the zinc ammonium sulfate hexahydrate salt comprises a zinc atom (gray sphere) coordinated to six water molecules [Zn(H_2_O)_6_] in a slightly distorted octahedron. The hydrogen atoms present in the H_2_O molecules of the coordination compound act as donors of hydrogen bonds, while the oxygen atoms (red spheres) present in the [SO_4_]^2–^ tetrahedra participate as acceptors of O–H···O hydrogen bonds. The NH_4_^+^ tetrahedra participate in intermolecular contacts as hydrogen donors (white spheres) of the N–H···O type. Moreover, it is the molecular fragments of H_2_O, NH_4_^+^, and [SO_4_]^2−^ that are responsible for imparting structural and thermal stability to the crystalline lattice of Tutton salt NHZnSO. This is attributed to the intermolecular interactions among fragments within the unit cell (see Fig. 2c) that propagate in three dimensions, stabilizing the crystal and resulting in an increased lattice energy.

### Hirshfeld surfaces, 2D-fingerprint plots, and crystal void analyses

**Fig. 3:**
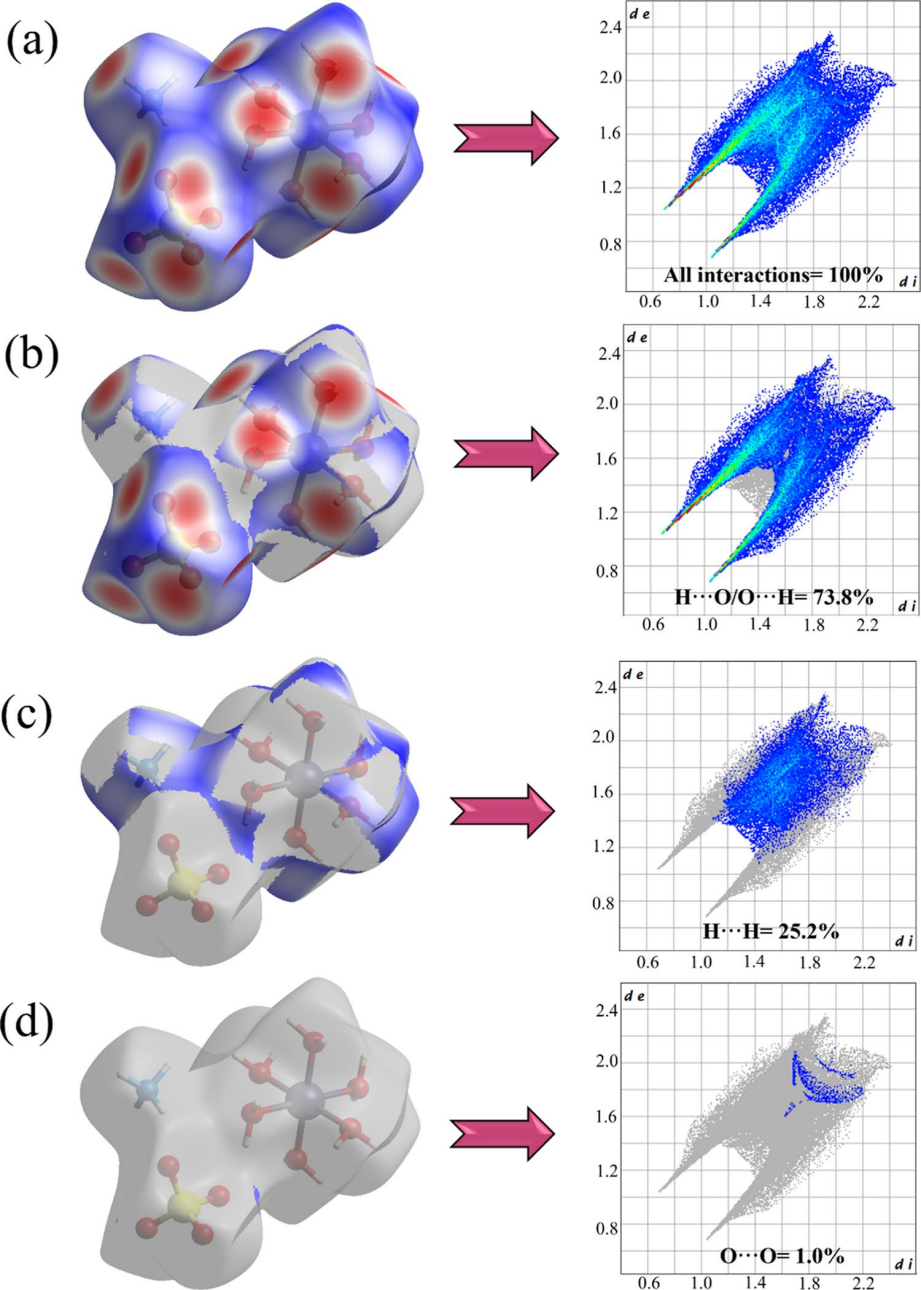
Hirshfeld surface mapping corresponding to *d*_norm_ (left) and 2D-fingerprint plots of interactions (right): cumulative (**a**), H···O/O···H (**b)**, H···H (**c**), and O···O (**d**)

A detailed structural analysis of the intermolecular interactions was carried out using Hirshfeld surfaces associated with 2D-fingerprinting, as shown in Fig. 3. The theoretical data were calculated from the crystal structure (ICSD-65013) previously solved by Montgomery and Lingafelter [26]. The color gradient in the 3D maps characterized the intensity of the contacts among chemical species: red, intermolecular interactions with distances shorter than *r*_dvW_; white, intermolecular interactions equivalent to the *r*_dvW_; blue, intermolecular interactions with distances greater than *r*_dvW_. As seen in Fig. 3a (left side), the red and white areas are distributed around the oxygen and hydrogen atoms present in the molecular fragments, with a strong predominance of H···O/O···H, H···H, and O···O interactions. Complementarily, the 2D-fingerprint plot (right side of Fig. 3a) displays the cumulative pattern involving all intermolecular interactions of the crystal lattice in terms of *d*_*e*_ and *d*_*i*_. The close contacts and distant contacts are represented by red and blue dots, respectively. In addition, the occurrence of sharp peaks in the regions of low* d*_*e*_ and *d*_*i*_ values indicates the interaction of strong contacts.

Figure 3b, c, and d present the Hirshfeld surface maps with their corresponding 2D-fingerprint plots. The stratified histograms show that the H···O/O···H contact, in addition to presenting the highest percentage contribution (73.8%) in the crystal structure, is also the most intense and originates from H_2_O and [SO_4_]^2–^ moieties. This observation is supported by the widespread distribution of points across most of the histogram and by elongated red peaks in areas with low *d*_e_ and *d*_i_ values. The H···H interaction also exhibits significant contributions, totaling around 25.2%, originating from H_2_O and NH_4_^+^ moieties. Weaker dispersive interactions (O···O) were also identified, with a percentage of around 1.0%, describing contacts of an induced dipole nature. In summary, the hydrogen bonds (H···O/O···H) are the main intermolecular interactions, promoting most structural stability to the crystalline system of Tutton salts, as also observed for crystals of (NH_4_)_2_Mn(SO_4_)_2_(H_2_O)_6_ (72.7%) [4], K_2_Mn_0_._03_Ni_0.97_(SO_4_)_2_(H_2_O)_6_ (59.0%) [43], and (NH_4_)_2_Cu(SO_4_)_2_(H_2_O)_6_ (69%) [23]. The variations in the percentage contribution of H···O/O···H contacts among these materials are attributed to differences in their coordination spheres and physicochemical properties, such as atomic radius, electronegativity, and electron affinity.

**Fig. 4:**
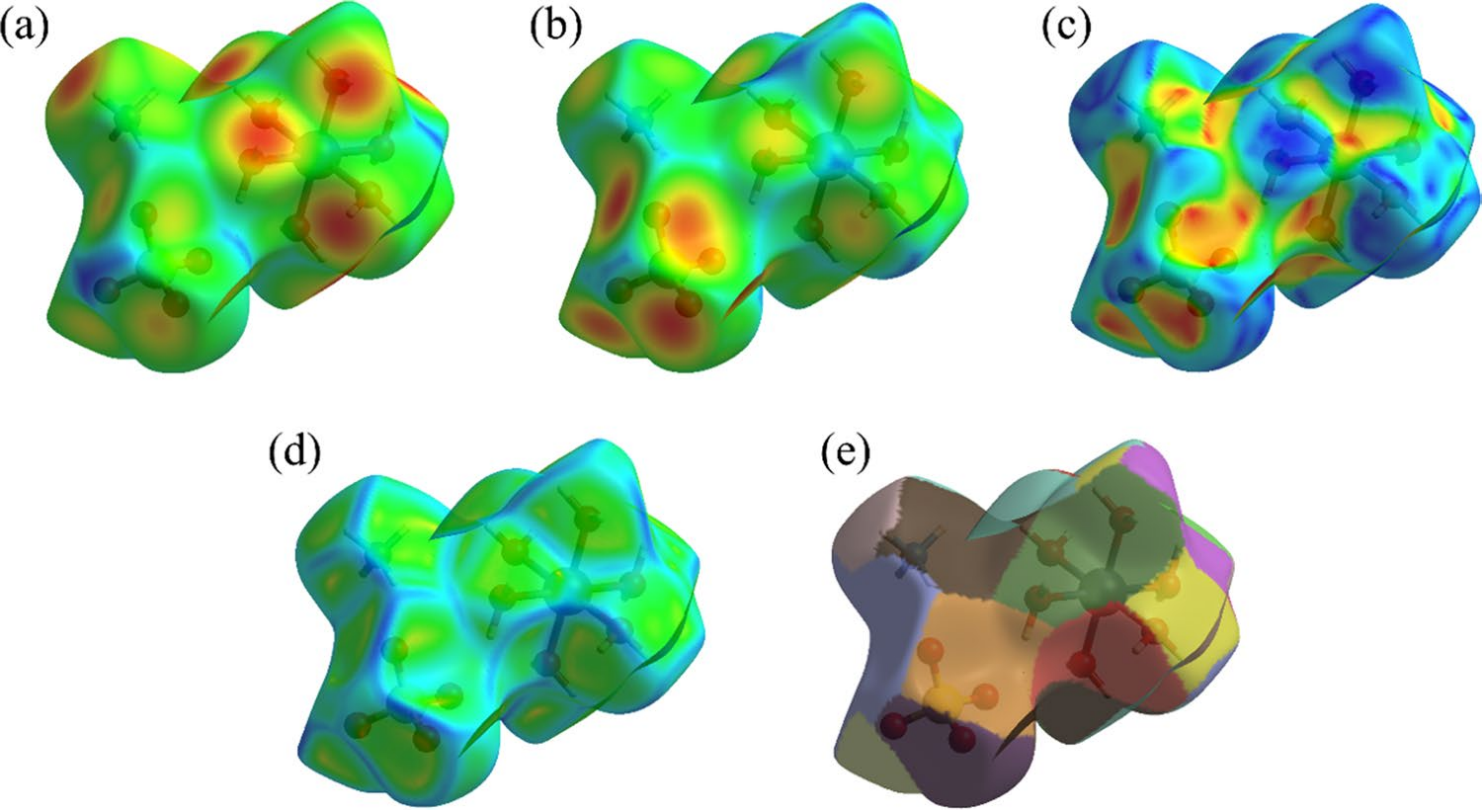
Hirshfeld surface mapping according to* d*_i_ (**a)**, *d*_e_ (**b**), shape index (**c**), curvedness (**d**), and fragment patch (**e**)

The Hirshfeld surfaces mapped in terms of *d*_i_ and *d*_e_ are shown in Fig. 4a and b, respectively. The reddish and yellowish regions around the oxygen atoms of the H_2_O ligands, and the hydrogen atoms of NH_4_^+^ shown in Fig. 4a characterize sites that act as donors in intermolecular interactions, while in Fig. 4b the same colors around the oxygen atoms from the [SO_4_]^2−^ group describe contacts that act as receptors of intermolecular interactions. Additionally, the shape index (Fig. 4c) and curvedness (Fig. 4d) of the surfaces shown are related to topological contacts used to identify the packing of molecular layers in the structural lattice via planar stacking. The red-toned zones presented in Fig. 4c, for example, are correlated with concave regions and the blue-toned zones represent convex regions. From this colored representation of shape, it is possible to visualize how adjacent species come into contact with each other [44]. For example, in the reddish areas stacking occurs with the neighboring molecular units through shorter and more intense contacts, while in the bluish areas, these contacts are more distant. The curvedness representation in Fig. 4d provides further insight into the interaction preferences between molecular moieties, in which the blue edges delimit the intermolecular contacts and exhibit high curvature values, while the greenish flat regions characterize low curvature values on the surface. Complementarily, the fragment patch regions projected on the surface of Fig. 4e are used to indicate the areas, in which neighboring molecules are stacked together in the atomic ordering that propagates throughout the crystal lattice [45].

Indeed, the shape index, curvedness, and fragment patch surfaces shown in Fig. 4c–e are complementary computational analyses that can be used to identify particular atomic packing modes and how adjacent moieties come into contact with each other [33]. For example, the shape index in Fig. 4c clearly shows that the reddish regions around the oxygen atoms of the [SO_4_]^2−^ group are conducive to the intermolecular interactions, and as the curvedness plots (Fig. 4d) show flat surfaces with spots, this highlights a favorable zone for planar stacking with neighboring moieties. Furthermore, the two regions highlighted in the tetrahedron in Fig. 4e in different colors suggest the locations where nearby units can fit together.

Another computational methodology employed to provide structural information about the physical properties of the unit cell is crystal voids. Using electron density isosurfaces with an area of 239.09 Å^2^, the lattice vacancies were identified, in which a percentage of 8.53% of empty spaces was found within the primitive cell, which corresponds to a volume of approximately 60.19 Å^3^. These data indicate that the NHZnSO crystal has voids available for the introduction of impurities to obtain or improve a specific property. Furthermore, void percentage values below 15% are associated with high lattice energies (potential energies that relate to the stability of ionic species) among the molecular units [22], suggesting stabilization of molecular moieties in the crystal solid.

### Geometry optimization

**Fig. 5:**
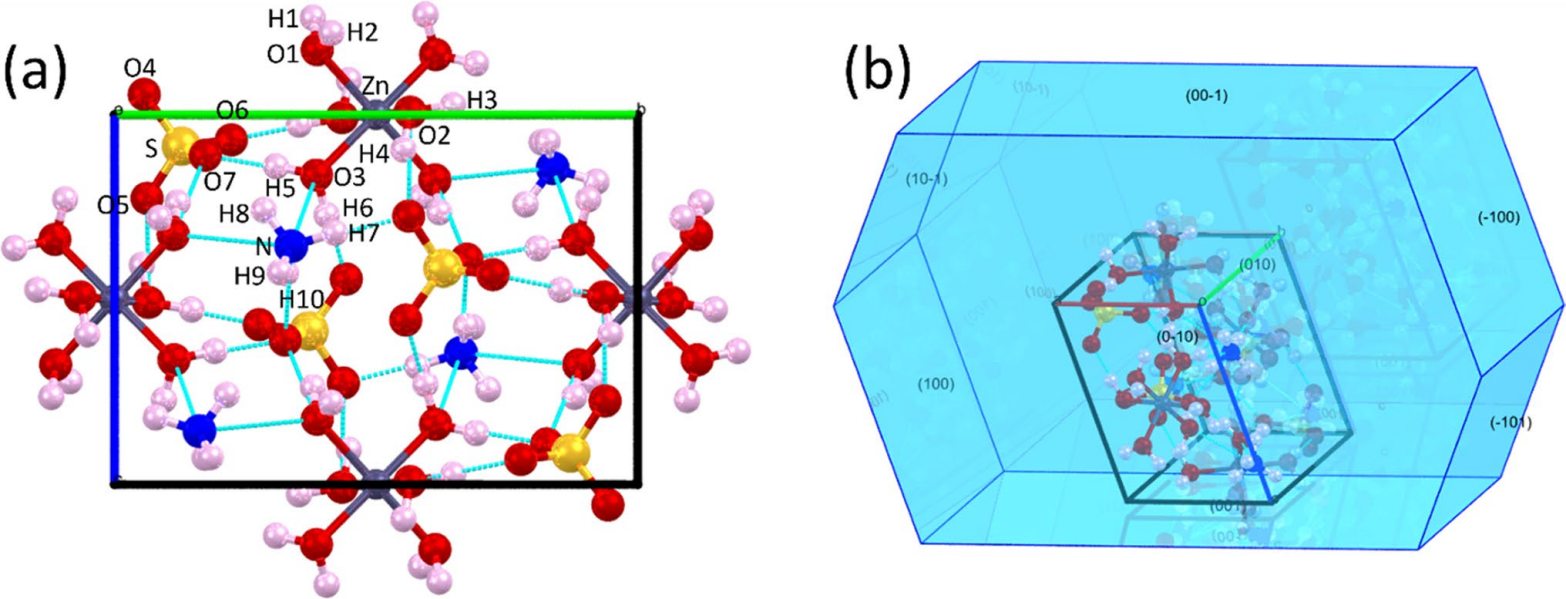
**a** Optimized unit cell of the NHZnSO Tutton salt obtained with the GGA-PBE functional using the DFT method. **b** Optimized morphology of NHZnSO crystal showing the crystalline planes and growth habit

From the DFT calculations, the structure of NHZnSO Tutton salt was duly optimized (see Fig. 5a) through the primitive unit cell based on the DFT method. Table 1 presents the relaxed cell parameters, compared with previously published experimental values [31] and with data extracted by the Rietveld method. As observed, all lattice parameters exhibit good agreement with each other. Furthermore, a comparative study was carried out involving calculated and experimental bond lengths and angles, as shown in Table 2.

**Table 1:** Optimized lattice parameters computed using the DFT method compared with the lattice parameters from the literature [30] and calculated from the Rietveld method for the NHZnSO Tutton salt

Structural parameters	Literature	Rietveld	DFT
*a* [Å]	9.236	9.343	9.236
*b* [Å]	12.514	12.648	13.515
*c* [Å]	6.246	6.241	6.246
α = γ [°]	90.00	90.00	90.00
β [°]	106.84	109.92	106.84

**Table 2:** Geometric parameters of NHZnSO Tutton salt: bond lengths [Å] and bond angles [◦] determined from X-ray diffraction [30] and computed by the DFT method

Bond lengths [Å]			Bond angle [°]		
Bond type	Exp	Calc	Bond type	Exp	Calc
Zn–O1	2.11	2.07	O1–Zn–O2	90.77	89.10
Zn–O2	2.06	2.01	O2–Zn–O3	89.60	88.63
Zn–O3	2.10	2.04	O3–Zn–O1	88.80	87.90
S–O4	1.46	1.46	O4–S–O5	109.45	109.40
S–O5	1.48	1.48	O5–S–O6	108.08	107.83
S–O6	1.48	1.49	O6–S–O7	108.96	108.53
S–O7	1.48	1.48	O7–S–O4	110.77	111.13
N–H7	0.88	1.05	H7–N–H8	110.60	109.80
N–H8	0.83	1.04	H8–N–H9	110.00	109.92
N–H9	0.97	1.05	H9–N–H10	106.70	106.30
N–H10	0.80	1.04	H10–N–H7	109.30	108.88
O1–H1	0.66	0.99	H1–O1–H2	104.40	109.09
O1–H2	0.78	0.99	H3–O2–H4	104.40	105.17
O2–H3	0.83	1.00	H5–O3–H6	104.40	107.01
O2–H4	0.73	0.99			
O3–H5	0.82	0.99			
O3–H6	0.91	1.00			

The DFT calculations were developed considering the propagation of the primitive unit cell in the reciprocal space with its corresponding intermolecular interactions. In this case, the calculations involved contacts between neighboring units since the methodology simulates the condensed phase. This fact justifies the good correlation between the experimental and theoretical data presented in Table 2. Based on the geometric parameters obtained, these results indicate that GGA-PBE is a suitable method for an accurate analysis of the structural, electronic, and vibrational properties of Tutton salts, as previously observed in other works of this nature [25].

In addition to the optimized geometry, it was possible to estimate through DFT calculations that the NHZnSO crystals have a lozenge-like shape as shown in Fig. 5b. This result agrees with the monoclinic symmetry (*P*2_1_/*a*) crystal structure experimentally obtained by the slow solvent evaporation method (see Fig. 1). Additionally, our results show that this system has eight crystallographic planes: $(\overline{1 } 0 0)$, $(0 \overline{1 } 0)$, $(0 0 1)$, $(\overline{ 1 } 0 1)$, $(0 0 \overline{1 })$, $(1 0\overline{ 1 })$, $(0 1 0)$, and $(1 0 0)$. However, during the solid phase crystallization process, not all planes are favored, due to the lack of control over the evaporation rate, which can induce the appearance of defects in the morphology.

### Electronic properties

**Fig. 6:**
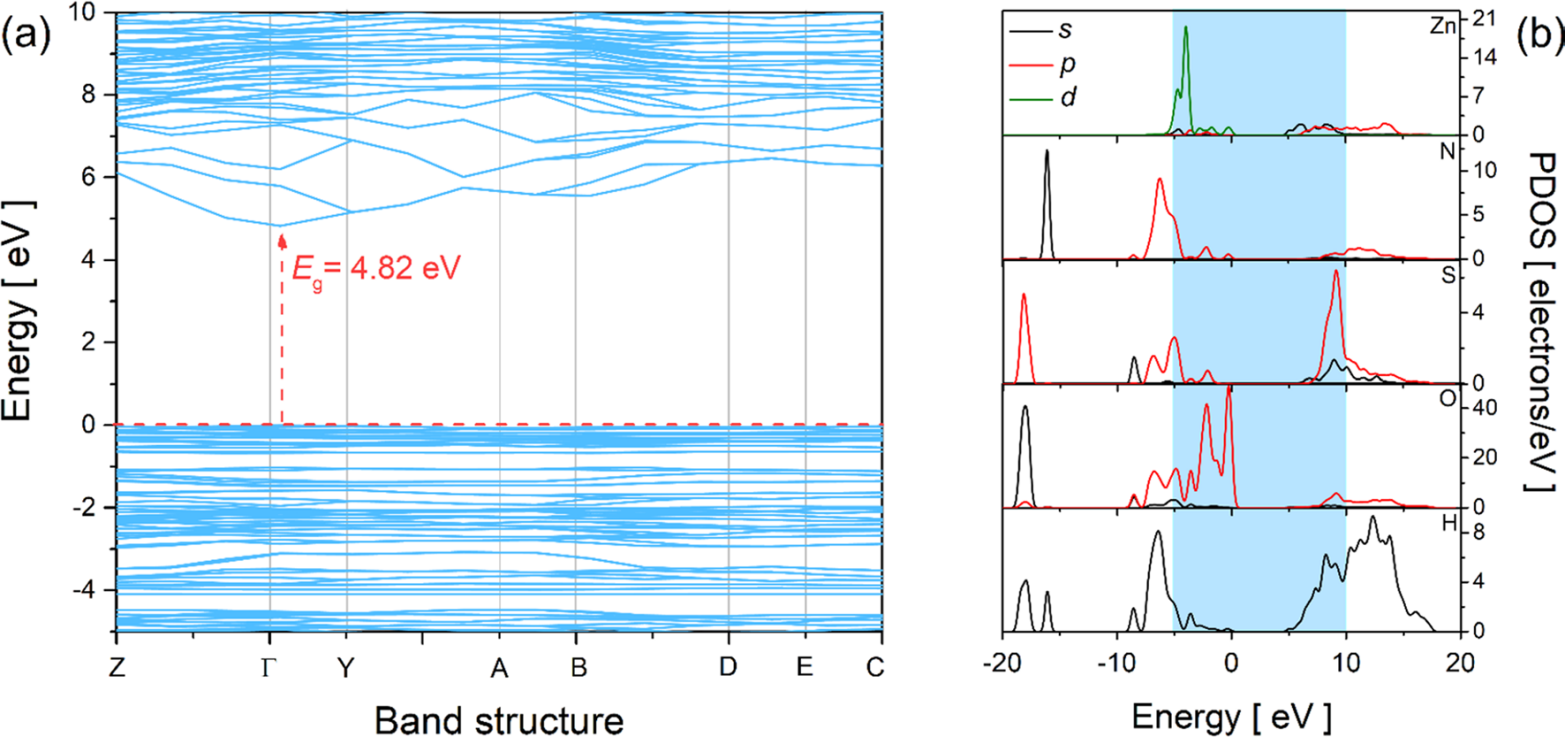
**a** Band structure of the Tutton NHZnSO salt. **b** Contributions of *s*, *p*, and *d* orbitals around the bandgap region from the PDOS

The electronic properties of the NHZnSO crystal were explored using the band structure and projected density of states (PDOS). Figure 6a presents the band structure from the high symmetry points that represent the limits of the first Brillouin zone, identified as the central regions Γ, Y, A, B, D, and E, as a function of energy. As verified, there are two distinct sets of bands: (I) Energy > 0, composed of wide bands in the upper part; (II) Energy < 0, composed of narrow bands at the bottom. The gap between the valence band and the conduction band, located at Γ, results in a direct energy bandgap of 4.82 eV. This value characterizes the dielectric nature of the NHZnSO system, as observed in the energy band gap experimentally obtained from the absorption coefficient. The slight variation observed between the experimental and calculated data can be attributed to the thickness of the sample and the defects present in the crystal. These defects spontaneously arise on the crystal surface due to uncontrolled solvent evaporation rates, leading to disruptions in the crystalline lattice regularity and the formation of surface dislocations. Furthermore, the close agreement observed between the theoretical and experimental results suggests that the PBE functional performs well in predicting the electronic nature of hydrated double salts. Moreover, the bandgap calculated herein is slightly higher than that reported for the Tutton K_2_Zn(SO_4_)_2_(H_2_O)_6_ salt, which exhibits a bandgap of 4.66 eV [24] and is similar or lower than the bandgaps of (NH_4_)_2_ M*(SO_4_)_2_(H_2_O)_6_ (M* = Ni, Cu, and Mn), which range from 4.80 to 6.07 eV [23, 44]. Although the bandgap values vary, all listed here refer to dielectric materials. These variations can be attributed not only to structural and compositional differences but also to functionals and other input parameters employed in the DFT calculation.

Figure 6b shows the PDOS distribution in the valence band around 20 electrons/eV for the Zn, O, S, N, and H atoms. The main contributions of Zn are from the *d* orbital, with only minor contributions from the *s* and *p* orbitals. For the O atom, a maximum density close to 45 electrons/eV was detected for the *s* and *p* orbitals. While for S and N atoms, the maximum values reached were 6 (*p* orbital) and 12 (*s* orbital) electrons/eV, respectively. Furthermore, for the H atom, only the contribution of the *s* orbital is around 9 electrons/eV, as expected. According to the results, all elements present in the NHZnSO crystal contribute *s* and *p* orbitals to the valence band.

The experimental UV–Vis absorbance and transmittance spectra recorded at ambient conditions for the NHZnSO single crystal are presented in Fig. S1. As it can be observed, no absorbance and transmittance bands are detected in the visible region, due to the electronic configuration of the Zn(II) ion within the [Zn(H_2_O)_6_]^2+^ coordination sphere. This behavior arises due to all *d* orbitals in the transition metal being completely filled (the electronic configuration of Zn^2+^ is *d*^10^), and the absence of electronic transitions allowed to absorb light in the visible region, justifying the colorless character of the single crystal, as presented in Fig. 1. Besides that, the H_2_O ligands are not chromophores in the UV–Vis region. This behavior suggests that NHZnSO could serve as a host matrix for embedding luminescent species in light-emitting devices. In addition, the fundamental absorption property was also evaluated. The absorption coefficient (α), which measures the attenuation of the light intensity that passes through crystal, is determined based on the following relationship [18]:

2 $$\alpha \left(\lambda \right)= \frac{1}{d}ln\left[\frac{1-R(\lambda ){)}^{2}}{T(\lambda )}\right],$$

where *d* is the crystal thickness, *R* is the reflectance, and *T* is the transmittance wavelength of the sample. As the reflectance signal in our experiments is very low, due to the high transmittance, *R* = 0 is assumed. The result is presented in the inset of Fig. S1 (Supplementary Material), where the value of *E*_g_ is extrapolated from the linear portion in the abscissa data at *α*(*λ*) = 0. The value of *E*_g_ shown in the inset of Fig. S2 is directly related to Eq. (2), as the estimated band gap (4.48 eV) corresponds to the excitation of electrons from the valence band to the conduction band and indicates the insulating nature of the material. This experimentally determined band gap is in a good agreement with the band gap calculated using DFT and presented in Fig. 6a.

### Group theory and vibrational analyses

As previously discussed, the Tutton NHZnSO salt is formed from three moieties co-crystallized in the crystal structure: NH_4_^+^, [SO_4_]^2–^, and [Zn(H_2_O)_6_]^2+^, in which they have 10, 10, and 19 atoms, respectively, within the unit cell. As there are 2 formulas [(NH_4_)_2_Zn(SO_4_)_2_(H_2_O)_6_] in the primitive cell, the crystal displays 78 atoms that are associated with 234 degrees of freedom, described according to the following irreducible representations for the *C*_*2h*_-factor group: Γ^total^ = 57A_u_ + 60A_g_ + 57B_u_ + 60B_g_, where the fundamental modes for IR activity are represented by A_u_ and B_u_, whereas for Raman activity are A_g_ and B_g_ [47]. Among the 234 vibrational modes, A_u_ + 2B_u_ (Γ^ac^ = A_u_ + 2B_u_) correspond to acoustic modes; therefore, the number of optically active modes is equivalent to Γ^Raman^ = 60A_g_ + 60B_g_ and Γ^IR^ = 57A_u_ + 57B_u_.

**Fig. 7:**
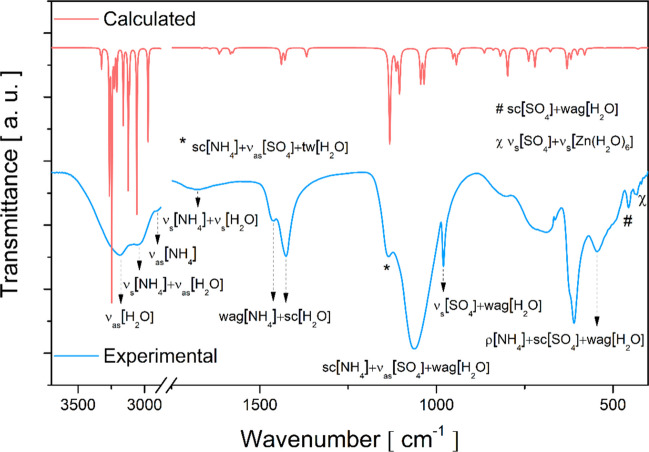
Experimental and calculated IR spectra of the Tutton NHZnSO salt: in the 3600–400 cm^–1^ region

**Fig. 8:**
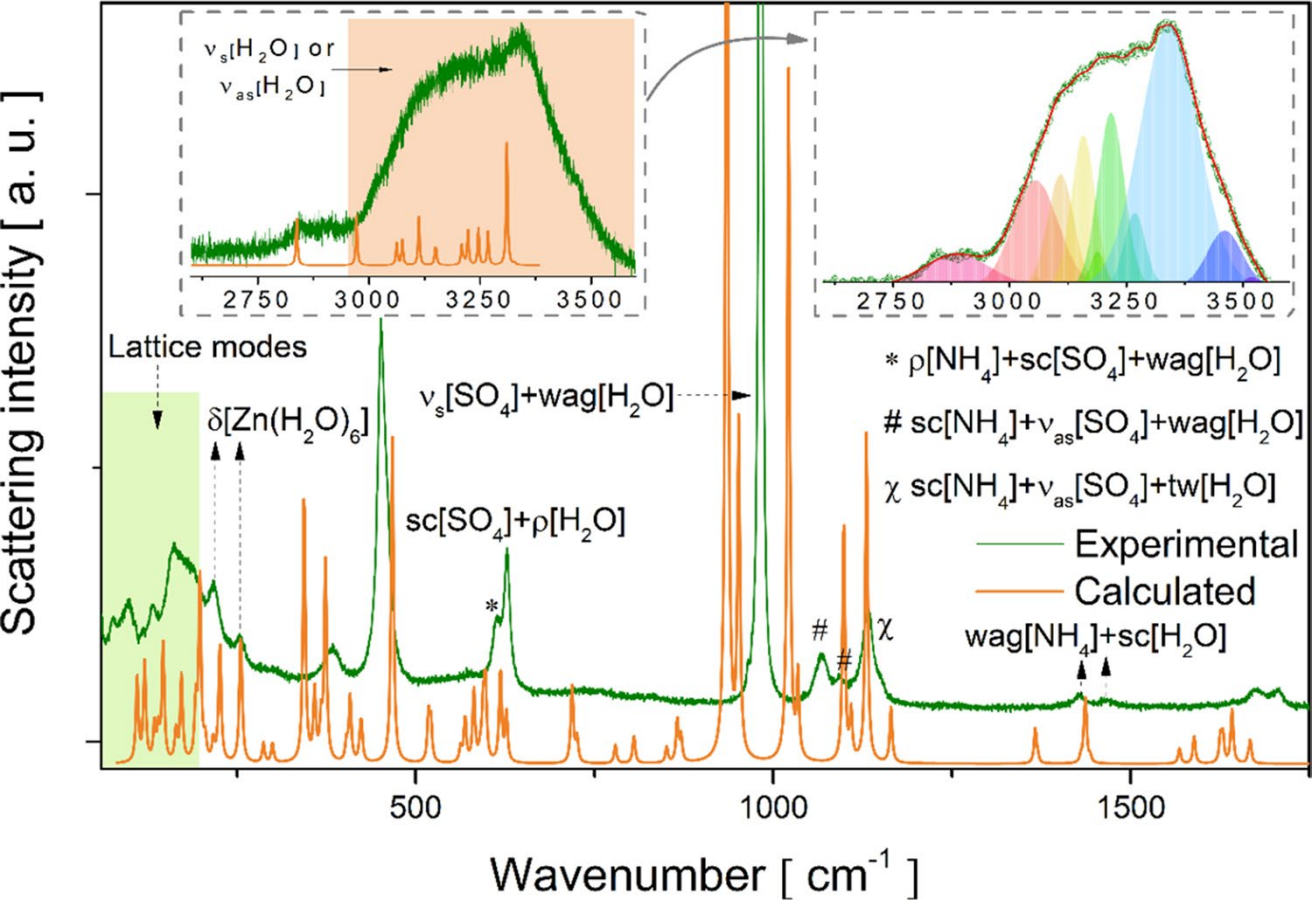
Experimental and calculated Raman spectra of the Tutton NHZnSO in 60–3600 cm^–1^ region, with inset showing Lorentzian deconvolution to twelve characteristic modes

Figures 7 and 8 show the experimental and calculated IR and Raman spectra, respectively. The band positions, irreducible representations, and assignments associated with each observed mode are recorded in Table S2 (Supplementary Material). In the 3600–2900 cm^−1^ spectral range of the IR absorption spectrum, a very broad band is observed, in which two modes are recorded at 3184 and 3045 cm^−1^ associated with the anti-symmetric stretching of H_2_O molecules, and one mode at 2904 cm^−1^ corresponding to the fingerprint anti-symmetric stretching of the NH_4_^+^ tetrahedron. In this same interval in the Raman spectrum (see insets of Fig. 8), a broad band was also found, in which, from a Lorentzian deconvolution, twelve characteristic modes of symmetric and anti-symmetric stretching vibrations (shown in the inset of Fig. 8) of the coordinated H_2_O molecules were identified. Furthermore, it was analyzed that the H_2_O modes were present in all spectral ranges, due to water acting as the main fragment that stabilizes the crystal lattice of Tutton salts, through a complex scheme of intermolecular contacts. Therefore, at shorter wavelengths, there were several contributions from water, such as scissoring, wagging, rocking, and twisting, in both spectra. This behavior is also observed in organic compounds that have a hydroxyl (OH) or carboxyl (COOH) group, which exhibit predominant vibration modes in the IR and Raman spectra and are the units responsible for connecting the main molecule with its surroundings [48–52].

Additionally, four bands were observed in the IR spectrum ranging from 2900 to 1160 cm^−1^, properly described as modes of the NH_4_^+^ tetrahedron (symmetric stretching, twisting, and wagging) with minor contributions from H_2_O molecule modes. Correspondingly, similar modes were detected in the Raman spectrum at ≈ 2886, 1645, and 1428 cm^−1^. Other equivalent modes can be found in Table S2.

In addition, the bands positioned at 627, 982, 1067, and 1149 cm^−1^ in the Raman spectrum were associated with characteristic contributions of the [SO_4_]^2−^ group interacting with the H_2_O molecules present in the [Zn(H_2_O)_6_]^2+^ coordination compound, as observed for hydrated salts K_2_Ni(SO_4_)_2_(H_2_O)_6_ [16], K_2_Mn(SO_4_)_2_(H_2_O)_2_ [18], and K_2_Zn(SO_4_)_2_(H_2_O)_6_ [25]. In the IR spectrum, the corresponding modes were observed at around 622, 808, and 979 cm^−1^. Furthermore, in the spectral range between 1150 and 1000 cm^−1^ for both IR and Raman spectra, scissoring and stretching vibrational modes were analyzed related to the NH_4_^+^ and [SO_4_]^2−^ tetrahedra, respectively, with contributions from the wagging or twisting vibrations of H_2_O molecules. Complementarily, other bands at shorter wavelengths (615–450 cm^−1^) correspond to characteristic vibrations of the NH_4_^+^, [SO_4_]^2−^, and H_2_O molecular fragments.

In the low wavenumber region of the IR spectrum, around 440–400 cm^−1^, three weak absorption bands corresponding to symmetric or anti-symmetric stretching vibrations of the [Zn(H_2_O)_6_] complex with contributions from NH_4_^+^ and [SO_4_]^2−^ vibrational modes were observed. Indeed, Tutton salts present IR bands of weak intensity below 450 cm^−1^, as previously reported in other works [46, 53]. Furthermore, similar modes were also identified in the Raman spectrum around 216, 254, 298, and 382 cm^−1^, related to the stretching or bending modes of the hexahydrate complex and the tetrahedra.

On the other hand, in the shorter wavelength region of the Raman spectrum, below 200 cm^−1^, ten bands were detected and correlated to the intermolecular modes of the lattice. According to DFT calculations, these were assigned to translational motions of the NH_4_^+^ tetrahedron combined with deformations or twists of the [SO_4_]^2−^ and [Zn(H_2_O)_6_] fragments, as presented in Table S2.

### Thermal behavior investigated by TG–DTA, DSC, and PXRD

**Fig. 9:**
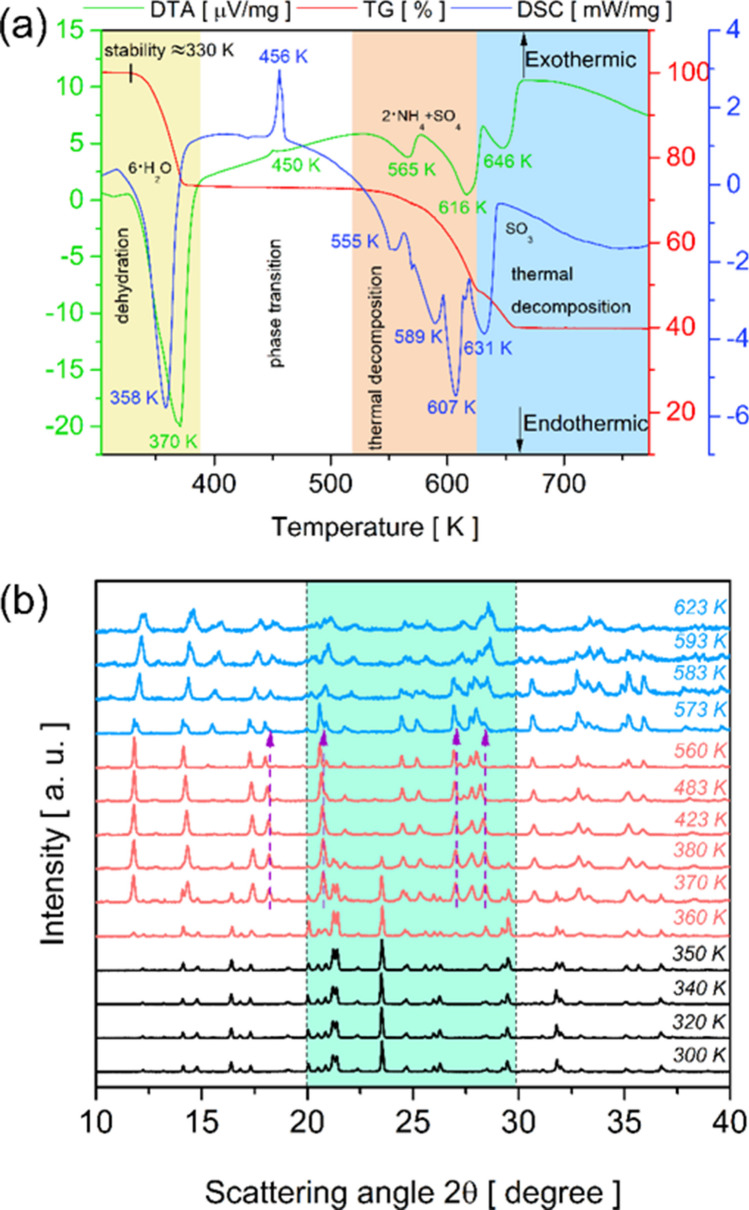
**a** TG–DTA, and DSC thermograms of the NHZnSO crystal in powder form. **b** Evolution of PXRD as a function of temperature for Tutton NHZnSO salt

The TG–DTA coupled and DSC thermograms in the temperature range between 300 and 773 K are presented in Fig. 9a. From the TG curve, it can be seen that the NHZnSO crystal is stable up to approximately 330 K. However, above this temperature, mass changes occur related to the degradation of the Tutton salt molecular fragments. The first mass loss (− 1.31 mg) occurs between 330 and 383 K and is associated with the release of six H_2_O molecules from the Zn(II) aqua complex (region highlighted in yellow, as shown in Fig. 9a), as follows: (NH_4_)_2_Zn(SO_4_)_2_(H_2_O)_6_ $\stackrel{\Delta }{\to }$ (NH_4_)_2_Zn(SO_4_)_2_. This event is accompanied by a broad endothermic peak located around 358 K and 370 K in the DSC and DTA curves, respectively, which characterizes total dehydration. Moreover, this process occurs with an enthalpy change of 734.56 kJ/mol, which is 122.43 kJ/mol per molecule of H_2_O. Kooijman et al. [3] reported a much lower value (63 kJ/mol per molecule of H_2_O) for the same double salt. The difference can be attributed to physical and instrumental factors, such as the amount of sample, heating rate, N_2_ gas flow rate, equipment model, and dehydration temperature of ≈ 366 K.

From the enthalpy value estimated by the DSC thermogram, the energy density of the NHZnSO crystal was calculated using Eq. (3), to verify the possibility of applying this salt in thermochemical energy storage systems:

3 $${\Delta H}_{v}= {\Delta H}_{m}+ {\rho }_{TS}$$

where, ${\Delta H}_{m}$ is the mass storage density $\left(\Delta {H}_{m}= \frac{\Delta H}{{MM}_{TS}}\right)$, $\Delta H$ is the value of experimental enthalpy (734.56 kJ/mol), ${MM}_{TS}$ is the molecular mass of the Tutton salt (401.65 g/mol), and ${\rho }_{TS}$ is the specific density (1.93 g/cm^3^). Therefore, the calculated energy density value was approximately 3.75 GJ/m^3^. This data makes Tutton NHZnSO salt a promising material for use in thermochemical materials, due to its dehydration temperature being lower than 393 K and energy density higher than 1.3 GJ/m^3^, which are key thermochemical energy storage material criteria [54]. Furthermore, our results show significantly better energy density compared with other salts previously reported in the literature [3], such as K_2_Zn(SO_4_)_2_(H_2_O)_6_ (1.79 GJ/m^3^) and Cs_2_Zn(SO_4_)_2_(H_2_O)_6_ (1.73 GJ/m^3^). These promising results corroborate the conclusions made by Kooijman et al. [3], who evaluated 24 hydrated double salts and highlighted that only (NH_4_)_2_Zn(SO_4_)_2_(H_2_O)_6_ has the high energy density and meets all the criteria required for use in heat-storing thermochemical devices.

At higher temperatures (> 383 K), the anhydrous NHZnSO salt presents a decay of TG curve in the range between 520 and 630 K (region highlighted in orange, as shown in Fig. 9a), which corresponds to a mass loss around − 1.66 mg, equivalent to 134.23 g/mol. This degradation process is related to the decomposition of two NH_4_^+^ moieties and a (SO_4_)^2−^ group, according to the chemical reaction (NH_4_)_2_Zn(SO_4_)_2_ $\stackrel{\Delta }{\to }$ ZnSO_4_. These decomposition stages of inorganic compounds are in agreement with published works associated with ammonium Tutton salts [2, 15, 23]. Moreover, above 630 K (region highlighted in blue, as shown in Fig. 9a) a final mass loss is detected in the TG curve of 0.98 mg (79.97 g/mol) associated with a SO_3_ molecule based on reaction ZnSO_4_ $\stackrel{\Delta }{\to }$ ZnO.

The endothermic events presented in the DTA and DSC curves in this temperature range (> 383 K) confirm the decomposition of inorganic moieties in a sequence of steps, demonstrating a phase transformation. Generally, compounds that exhibit high molecular weight and a complex hydrogen bonding scheme tend to require more than one region for decomposition [55, 56]. However, before these endo-events, there is an exothermic peak at 450 K in the DTA curve and at 456 K in the DSC curve correlated with a structural change of the anhydrous phase as a function of the increase in heat rate. Table 3 provides a summary of the temperatures associated with the events and mass loss regarding the heating of the Tutton salt NHZnSO.

**Table 3:** Thermals events observed for the Tutton NHZnSO salt in TG–DTA and DSC analysis

Temperature range [K]	Compounds	TG			DTA T_peak_ [K]	DSC T_peak_ [K]
		Weight loss [mg]	Weight loss [%]	Molar mass [g/mol] *Calc* “Exp”		
330–383	6∙H_2_O	1.31	26.44	*106.20* “108.09”	↓ 370 (1*)	↓ 358 (1)
383–630	2∙NH_4_ + SO_4_	1.66	33.42	*134.23* “149.15”	↑ 450 (2*), ↓ 565 (3*), ↓ 616 (4*), and ↓ 646 (5*)	↑ 456 (2), ↓ 555 (3), ↓ 589 (4), ↓ 607 (5), and ↓ 631 (6)
> 630	SO_3_	0.98	19.73	*80.66* “79.97”	-	-

From the thermal results obtained through TG–DTA and DSC thermograms, PXRD measurements as a function of temperature were carried out to evaluate the structural behavior of NHZnSO with increasing heat rate. Figure 9b displays the diffractograms recorded between 300 and 623 K. As shown, up to 350 K (black lines, Phase 1) only physical parameters characteristic of Tutton salts are observed (monoclinic symmetry *P*2_1_/*a* (n° 14) (${C}_{2h}^{5}$) space group). However, subtle changes are observed around 340 K, such as shifts and broadening of peaks. This evidence can be better visualized in the intensities map detailed in Fig. S2 (Supplementary Material).

Above 360 K, more prominent structural changes are observed in the diffractograms presented in Fig. 9b (red lines), such as the appearance of peaks located around 2θ = 11.74°, 14.33°, and 18.22°. However, up to around 400 K, there is a mixture between the hexahydrate and anhydrous phase, as shown in Fig. S2 (Supplementary Material). Nevertheless, above 400 K, the anhydrous structure of NHZnSO salt appears well-defined and remains stable until 560 K. This new phase shows an expansion effect of the unit cell volume with increasing temperature, due to the displacement of peaks to smaller angles. Such modifications may be correlated with a structural change of the molecular fragments on the anhydrous phase, as inferred from the DTA and DSC results. The purple arrows on some peaks (2θ = 20.77°, 27.01°, and 28.41°) characterize this evidence.

To analyze the behavior of the anhydrous phase, the PXRD pattern at 483 K was chosen to determine the crystallographic parameters using the Le Bail method. The diffractogram was analyzed using DASH 3.4.5 software to index the peaks and identify the crystallographic structure. Then, the results were entered into the GSAS program for data refinement. Fig. S3 (Supplementary Material) shows the pattern at 483 K refined by the Le Bail method (with *R*_wp_ = 8.56%, *R*_p_ = 5.91%, and *S* = 1.69). As shown, the anhydrous phase (NH_4_)_2_Zn(SO_4_)_2_ exhibits monoclinic symmetry with P12_1_1 (n° 4) (${C}_{2}^{2}$) space group and the cell parameters: *a* = 18.282(4) Å, *b* = 13.731(9) Å, *c* = 14.179(2) Å, *α* = *γ* = 90.00°, and *β* = 102.12(6)°. The persistence of monoclinic symmetry in the anhydrous phase, even after the rupture of hydrogen bonds provided by H_2_O molecules, is due to the contacts established between the NH_4_^+^ and [SO_4_]^2−^ units. However, the change of space group *P*2_1_/*a* → *P*12_1_1 makes the atomic system more disordered.

In addition, after 560 K (see Fig. 9b), a new change in the PXRD patterns appears due to the initial decomposition of the NH_4_^+^ and [SO_4_]^2−^ moieties. Configuring a mixture of phases II and III up to around 583 K, as shown in the intensity map in Fig. S2 (Supplementary Material). Above this temperature, phase III begins its stabilization; however, up to the temperature range studied, it was not possible to resolve the structure of the ZnSO_4_ compound since at the maximum temperature analyzed, 623 K, the NH_4_^+^ and [SO_4_]^2−^ fragments did not undergo complete decomposition.

**Fig. 10:**
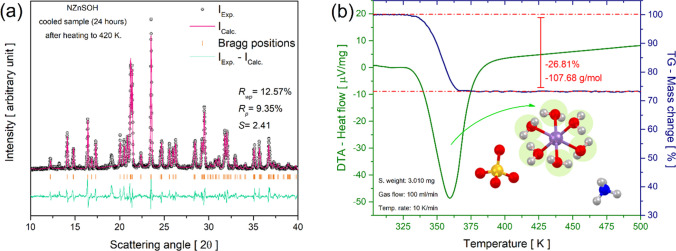
PXRD pattern refined by the Rietveld method (**a**) and TG–DTA curves after the sample was heated to 420 K and cooled at room conditions for 24 h (**b**)

To check whether this salt was cyclable, a powder sample was heated to 420 K in an oven and then cooled and left at room conditions for 24 h. Then, PXRD and TG–DTA measurements were performed to analyze the influence of the medium on the compound. The results are shown in Fig. 10a and b.

Figure 10a shows the PXRD pattern refined by the Rietveld method after the sample was heated to 420 K and cooled for 24 h at room conditions. The presented results indicate that NHZnSO undergoes rehydration when exposed to atmospheric air for 24 h after the dehydration. Under these conditions, the sample presented monoclinic symmetry and *P*2_1_/*a*-space group with the following refined cell parameters *a* = 9.259(3) Å, *b* = 12.556(4) Å, *c* = 6.251(6) Å, *α* = γ = 90.00°, *β* = 106.84(5)°, and V = 695.64(5) Å^3^, which characterize the Tutton salt phase. Furthermore, TG–DTA measurements were also performed and depicted in Fig. 10b. The TG curve indicated a mass loss of 26.81% (107.68 g/mol) between 328 and 373 K, which was associated with the release of approximately 6 H_2_O molecules coordinated to the Zn^2+^ center. The endothermic peak located around 360 K characterized the dehydration process of the double salt hexahydrate. The PXRD and TG–DTA techniques demonstrated that the NHZnSO crystal underwent rehydration in atmospheric air after 24 h of exposure.

Therefore, according to the criteria established for the application of materials in thermochemical energy storage devices: (*i*) dehydration temperature < 120 °C; (*ii*) energy density ≥ 1.3 GJ/m^3^; (*iii*) cyclability ≥ 10; (*iv*) hydration temperature > 50 °C; and (*v*) hydration vapor pressure < 12 mbar [43], the NHZnSO crystal becomes a promising system, as it meets at least three of the five defined requirements. However, broader studies of cyclability and vapor pressure should be carried out in the future.

## Conclusions

In this article, the NHZnSO crystal belonging to the Tutton salt family was successfully grown by the solvent evaporation method. The structural, spectroscopic, and thermal properties were presented and discussed. Computational studies based on the Hirshfeld surface and voids analysis methods were conducted. Furthermore, DFT calculations were employed to support the experimental results and obtain data associated with the electronic nature and bonding of the material.

At room conditions (300 K), the PXRD pattern refined by the Rietveld refinement method showed that the crystal structure was related to a Tutton salt, due to its monoclinic system with P2_1_/a space group. Additionally, through the analysis of Hirshfeld surfaces, the contacts that stabilize the crystalline lattice (H···O/O···H, H···H, and O···O) were identified and quantified. Moreover, an experimental bandgap of 4.48 eV was determined from the transmittance spectrum. Complementarily, using the band structure, a theoretical bandgap of approximately 4.82 eV was estimated, being directly influenced by the *s*, *p*, and *d* orbitals on the crystal valence band. These values agreed with each other and describe the insulating nature of NHZnSO Tutton salt. In addition, a good correlation between experimental and calculated data was observed for the IR and Raman spectra, allowing a detailed analysis of the vibrational modes.

The TG–DTA analysis showed that the double hexahydrate salt was stable up to about 330 K. Above this temperature, the crystal exhibits a phase transformation to the anhydrous form due to the dehydration of the six H_2_O molecules. Furthermore, around 450 K, the salt underwent a structure change in its dehydrated structure. At higher temperatures, > 520 K, inorganic compounds began a decomposition process. Complementarily, the results obtained from the DSC thermogram corroborated the data presented in the DTA curve. Moreover, it was possible to estimate the enthalpy of dehydration (122.43 kJ/mol of H_2_O) and the energy density of the material (3.75 GJ/m^3^). The PXRD results as a function of temperature confirmed the phase transformation of the crystal to the anhydrous form around 400 K. Using Le Bail’s method, it was verified that the new phase (NH_4_)_2_Zn(SO_4_)_2_ had monoclinic symmetry with P12_1_1 group space and was stable up to around 560 K. From these results it was possible to better understand the thermostructural properties associated with the temperature variation of the Tutton salt NHZnSO. The findings presented here indicated that the NHZnSO crystal exhibited thermostructural (low dehydration temperature, high energy density, and rehydration after 24 h) and electronic (high value of the energy gap) properties superior to those of crystals Na_2_Zn(SO_4_)_2_(H_2_O)_6_, K_2_Zn(SO_4_)_2_(H_2_O)_6_, Rb_2_Zn(SO_4_)_2_(H_2_O)_6_, and Cs_2_Zn(SO_4_)_2_(H_2_O)_6_, previously reported in the literature.

## Supplementary Information

Below is the link to the electronic supplementary material.

Supplementary file1 (DOCX 1024 KB)

## Data Availability

No datasets were generated or analysed during the current study.
