# Supplementary material for: Tutton salt (NH4)2Zn(SO4)2(H2O)6: thermostructural, spectroscopic, Hirshfeld surface, and DFT investigations
Source: J Mol Model. 2024 Sep 17;30(10):339. doi: 10.1007/s00894-024-06089-7 (PMC11408582; doi:10.1007/s00894-024-06089-7)
Supplement: Supplementary file 1 — Supplementary file1 (DOCX 1024 KB) [file 894_2024_6089_MOESM1_ESM.docx]

**Supplementary material**

**Tutton salt (NH_4_)_2_Zn(SO_4_)_2_(H_2_O)_6_: thermostructural, spectroscopic, Hirshfeld surface, and DFT investigations**

João G. de Oliveira Neto^1^ ∙ Jailton R. Viana^1^ ∙ Kamila R. Abreu^1^ ∙ Luiz F. L. da Silva^2^ ∙ Mateus R. Lage^1,*^ ∙ Stanislav R. Stoyanov^3,*^ ∙ Francisco F. de Sousa^1,4^ ∙ Rossano Lang^5^ ∙ Adenilson O. dos Santos^1^

1- Center for Social Sciences, Health, and Technology, Federal University of Maranhão - UFMA, Imperatriz, MA 65900-410, Brazil

2- Institute of Criminalistics, Scientific Police of Pará, Marabá, PA 68507-000, Brazil

3- Natural Resources Canada, CanmetENERGY Devon, 1 Oil Patch Drive, Devon, AB T9G 1A8, Canada

4- Institute of Exact and Natural Sciences, Federal University of Pará – UFPA, Belem, PA 66075-110, Brazil

5- Institute of Science and Technology, Federal University of São Paulo - UNIFESP, São José dos Campos, SP 12231-280, Brazil

*** Corresponding authors:** [mateus.lage@ufma.br](mailto:mateus.lage@ufma.br) (Mateus R. Lage); [stanislav.stoyanov@nrcan-rncan.gc.ca](mailto:stanislav.stoyanov@nrcan-rncan.gc.ca) (Stanislav R. Stoyanov).

**Table S1** Comparison between the structural parameters previously reported in the literature for the crystal (NH_4_)Zn(SO_4_)_2_(H_2_O)_6_ via single-crystal X-ray diffraction and the crystallographic data obtained in this study using the Rietveld method.

|  |  | **Literature** | **Present study** | **Difference [ % ]** |
| --- | --- | --- | --- | --- |
| **Structural parameters** | **Symmetry** | Monoclinic | Monoclinic | - |
|  | **Space group** | *P*2_1_/*a* | *P*2_1_/*a* | - |
|  | ***Z*** | 2 | 2 | - |
| **Lattice parameters** | ***a* [ Å ]** | 9.279(1) | 9.343(4) | 0.69 |
|  | ***b* [ Å ]** | 12.568(3) | 12.648(8) | 0.63 |
|  | ***c* [ Å ]** | 6.253(5) | 6.241(5) | 0.19 |
|  | **α [ ° ]** | 90.00 | **90.00** | **-** |
|  | **β [ ° ]** | 106.49(6) | 109.92(3) | 3.22 |
|  | **γ [ ° ]** | 90.00 | **90.00** | **-** |
|  | **V [ Å^3^ ]** | - | 705.65(9) | - |
| **Quality parameters** | ***R*_wp_ [ % ]** | **-** | 8.99 | - |
|  | ***R*_p_ [ % ]** | **-** | 5.48 | - |
|  | ***S*** | **-** | 1.65 | - |

**Table S2** Vibration mode analyses for the (NH_4_)_2_Zn(SO_4_)_2_(H_2_O)_6_ crystal: calculated wavenumbers (ωcal), experimental Raman modes (ωR), experimental IR modes (ωIR), irreducible representation (Irrep.), and their assignments.

| ωcal [ cm^-1^ ] | ω_R_ [ cm^-1^ ] | ω_IR_ [ cm^-1^ ] | Irrep | Assignments* |
| --- | --- | --- | --- | --- |
| 62.5 | 59 | - | A_g_ | trans[NH_4_] + δ[SO_4_] + τ[Zn(H_2_O)_6_] |
| 75.2 | 76 | - | A_g_ | trans[NH_4_] + δ[SO_4_] + τ[Zn(H_2_O)_6_] |
| 85.6 | 90 | - | B_g_ | trans_op_[NH_4_] + δ[SO_4_] + τ[Zn(H_2_O)_6_] |
| 100.4 | 97 | - | B_g_ | trans_op_[NH_4_] + δ[SO_4_] + τ[Zn(H_2_O)_6_] |
| 120.6 | 117 | - | B_g_ | trans[NH_4_] + δ[Zn(H_2_O)_6_] |
| 130.7 | 132 | - | B_g_ | trans[NH_4_] + δ[Zn(H_2_O)_6_] |
| 164.4 | 160 | - | B_g_ | δ[NH_4_] + tw[SO_4_] + δ[Zn(H_2_O)_6_] |
| 172.1 | 169 | - | A_g_ | δ[NH_4_] + tw[SO_4_] + δ[Zn(H_2_O)_6_] |
| 192.1 | 189 | - | B_g_ | trans[NH_4_] + δ[SO_4_] + δ[Zn(H_2_O)_6_] |
| 197.8 | 198 | - | A_g_ | trans[NH_4_] + δ[SO_4_] + δ[Zn(H_2_O)_6_] |
| 216.6 | 216 | - | B_g_ | δ[Zn(H_2_O)_6_] |
| 254.4 | 254 | - | A_g_ | δ_s_[Zn(H_2_O)_6_] |
| 299.4 | 298 | - | A_g_ | δ[SO_4_] + δ[Zn(H_2_O)_6_] |
| 373.5 | 382 | - | A_g_ | ν_s_[SO_4_] + ν_s_[Zn(H_2_O)_6_] |
| 398.1 | - | 403 | B_u_ | ν_s_[SO_4_] + ν_s_[Zn(H_2_O)_6_] |
| 427.8 | - | 419 | B_u_ | δ[NH_4_] + ν_s_[SO_4_] + ν_s_[Zn(H_2_O)_6_] |
| 431.5 | - | 432 | A_u_ | δ[NH_4_] + δ_s_[SO_4_] + ν_as_[Zn(H_2_O)_6_] |
| 466.9 | 451 | - | B_g_ | ρ[NH_4_] + sc[SO_4_] + wag[H_2_O] |
| 470.2 | - | 456 | A_u_ | sc[SO_4_] + wag[H_2_O] |
| 472.6 | - | 488 | B_u_ | sc[SO_4_] + wag[H_2_O] |
| 524.2 | - | 545 | A_u_ | ρ[NH_4_] + sc[SO_4_] + wag[H_2_O] |
| 603.6 | - | 610 | A_u_ | ρ[NH_4_] + sc[SO_4_] + wag[H_2_O] |
| 617.5 | 613 | - | B_g_ | ρ[NH_4_] + sc[SO_4_] + wag[H_2_O] |
| 623.9 | - | 622 | A_u_ | sc[SO_4_] + ρ[H_2_O] |
| 626.6 | 627 | - | A_g_ | sc[SO_4_] + ρ[H_2_O] |
| 676.2 | - | 663 | A_u_ | tw[H_2_O] |
| 678.3 | - | 690 | B_u_ | tw[H_2_O] |
| 721.0 | - | 718 | A_u_ | tw[H_2_O] |
| 725.2 | 741 | - | B_g_ | tw[H_2_O] |
| 801.3 |  | 808 | A_u_ | ρ[NH_4_] + ρ[H_2_O] |
| 805.5 | 819 | - | B_g_ | ρ[NH_4_] + ρ[H_2_O] |
| 870.9 | 884 | - | B_g_ | ρ[H_2_O] |
| 953.0 | - | 979 | B_u_ | ν_s_[SO_4_] + wag[H_2_O] |
| 954.8 | 982 | - | B_g_ | ν_s_[SO_4_] + wag[H_2_O] |
| 1034.3 | 1067 | - | B_g_ | sc[NH_4_] + ν_as_[SO_4_] + wag[H_2_O] |
| 1044.4 | - | 1062 | B_u_ | sc[NH_4_] + ν_as_[SO_4_] + wag[H_2_O] |
| 1098.3 | 1092 | - | A_g_ | sc[NH_4_] + ν_as_[SO_4_] + wag[H_2_O] |
| 1130.2 | 1132 | - | A_g_ | sc[NH_4_] + ν_as_[SO_4_] + tw[H_2_O] |
| 1133.1 | - | 1136 | A_u_ | sc[NH_4_] + ν_as_[SO_4_] + tw[H_2_O] |
| 1164.5 | 1149 | - | B_g_ | ν_as_[SO_4_] + wag[H_2_O] |
| 1429.0 | - | 1425 | A_u_ | wag[NH_4_] + sc[H_2_O] |
| 1430.0 | 1428 | - | A_g_ | wag[NH_4_] + sc[H_2_O] |
| 1441.9 | - | 1460 | A_u_ | wag[NH_4_] + sc[H_2_O] |
| 1443.3 | 1464 | - | B_g_ | wag[NH_4_] + sc[H_2_O] |
| 1625.0 | 1608 | - | A_g_ | tw[NH_4_] + sc[H_2_O] |
| 1640.5 | 1645 | - | B_g_ | tw[NH_4_] + sc[H_2_O] |
| 1663.6 | 1676 | - | B_g_ | tw[NH_4_] + sc[H_2_O] |
| 1664.9 | - | 1674 | B_u_ | tw[NH_4_] + sc[H_2_O] |
| 1666.9 | 1704 | - | A_g_ | tw[NH_4_] + sc[H_2_O] |
| 2837.1 | 2846 | - | A_g_ | ν_s_[NH_4_] + ν_as_[H_2_O] |
| 2840.4 | 2886 | - | B_g_ | ν_s_[NH_4_] + ν_as_[H_2_O] |
| 2844.9 | - | 2844 | A_u_ | ν_s_[NH_4_] + ν_as_[H_2_O] |
| 2971.8 | 2905 | - | A_g_ | ν_as_[NH_4_] + ν_s_[H_2_O] |
| 2973.5 | - | 2904 | B_u_ | ν_as_[NH_4_] |
| 3057.9 | - | 3045 | A_u_ | ν_s_[NH_4_]+ ν_as_[H_2_O] |
| 3062.0 | 3055 | - | A_g_ | ν_s_[H_2_O] |
| 3074.8 | 3059 | - | B_g_ | ν_s_[H_2_O] |
| 3112.0 | 3109 | - | A_g_ | ν_s_[H_2_O] |
| 3150.7 | 3157 | - | A_g_ | ν_as_[H_2_O] |
| 3158.3 | - | 3184 | A_u_ | ν_as_[H_2_O] |
| 3208.2 | 3187 | - | A_g_ | ν_as_[H_2_O] |
| 3246.0 | 3217 | - | A_g_ | ν_s_[H_2_O] |
| 3246.1 | 3268 | - | B_g_ | ν_s_[H_2_O] |
| 3267.4 | 3318 | - | A_g_ | ν_s_[H_2_O] |
| 3269.8 | 3341 | - | B_g_ | ν_s_[H_2_O] |
| 3310.0 | 3460 | - | A_g_ | ν_as_[H_2_O] |
| 3327.2 | 3519 | - | B_g_ | ν_s_[H_2_O] |

* Nomenclature: trans= translational; trans_op_= translational out-of-phase; τ= torsion; sc= scissoring; tw= twisting; wag= wagging; ν= stretching; ρ= rocking; δ= bending; δ_a_= anti-symmetric bending; δ_s_= symmetric bending; ν_a_= anti-symmetric stretching; ν_s_= symmetric stretching.

**
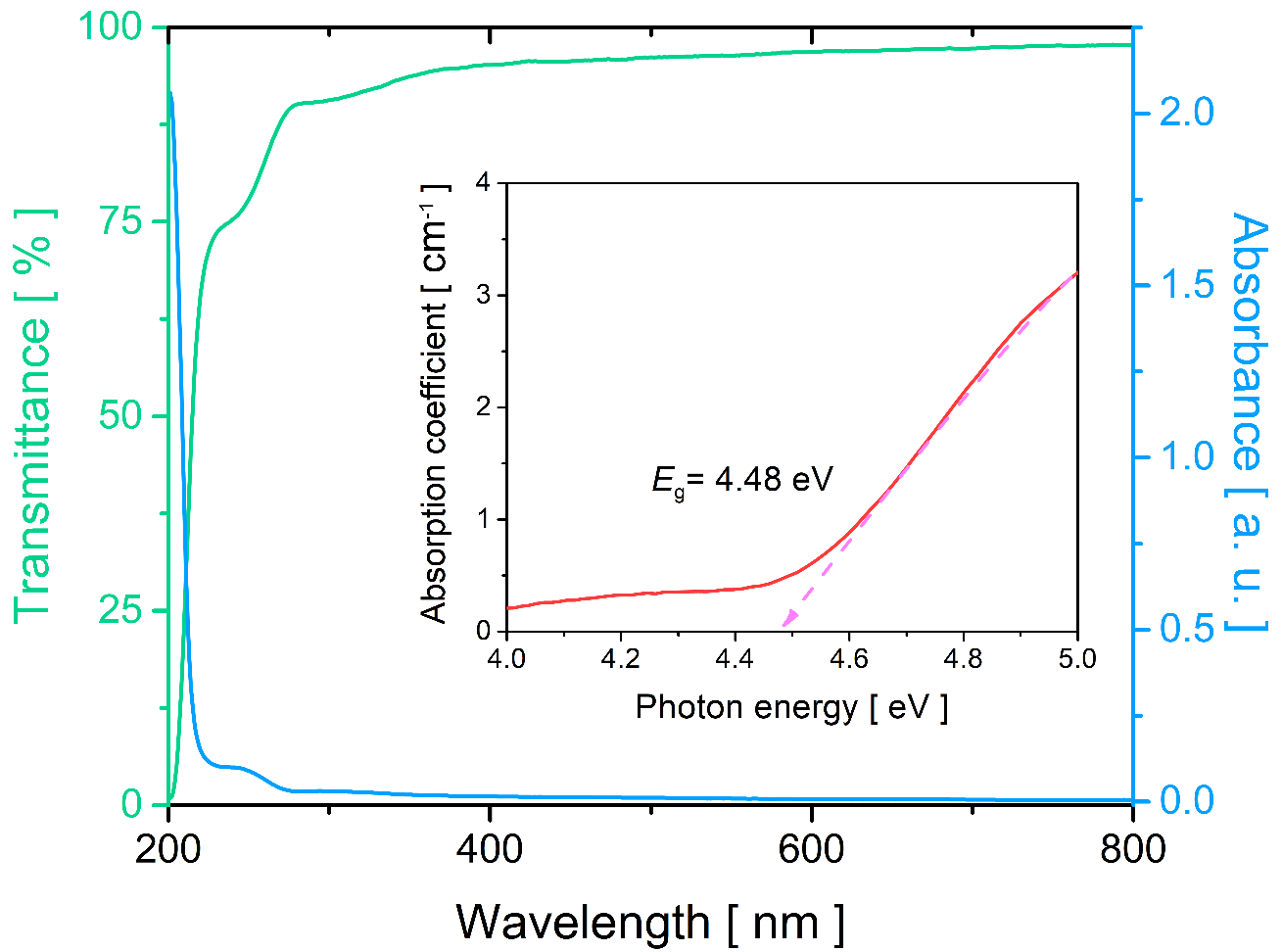
**

**Fig. S1** Experimental UV–Vis optical transmittance and absorbance spectrum of a NHZnSO single crystal. *Inset:* plot of the absorption coefficient as a function of photon energy, showing E_g_ as the x-intercept (dashed line).

**
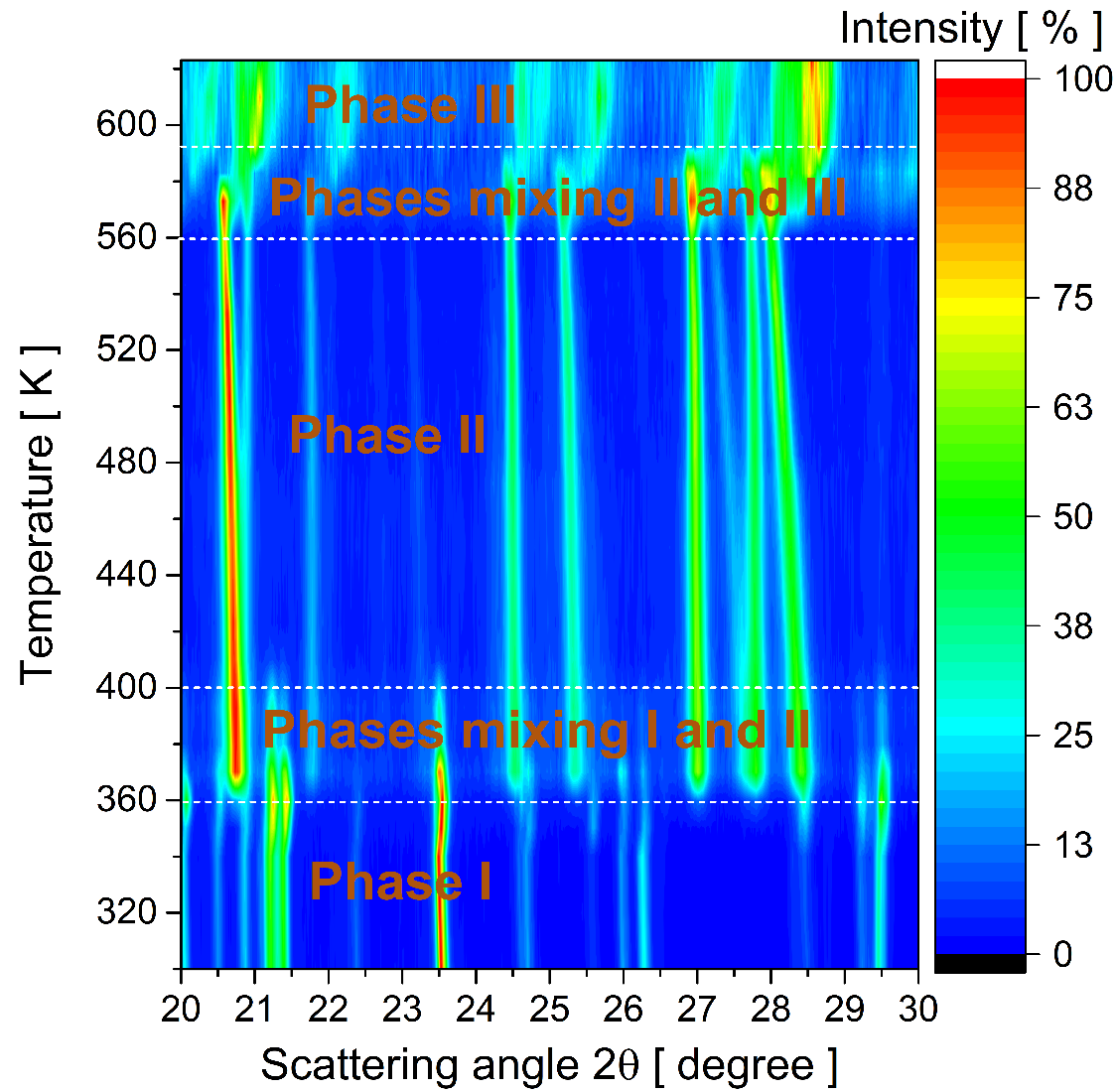
**

**Fig. S2** PXRD pattern intensity mapping as a function of temperature.

**
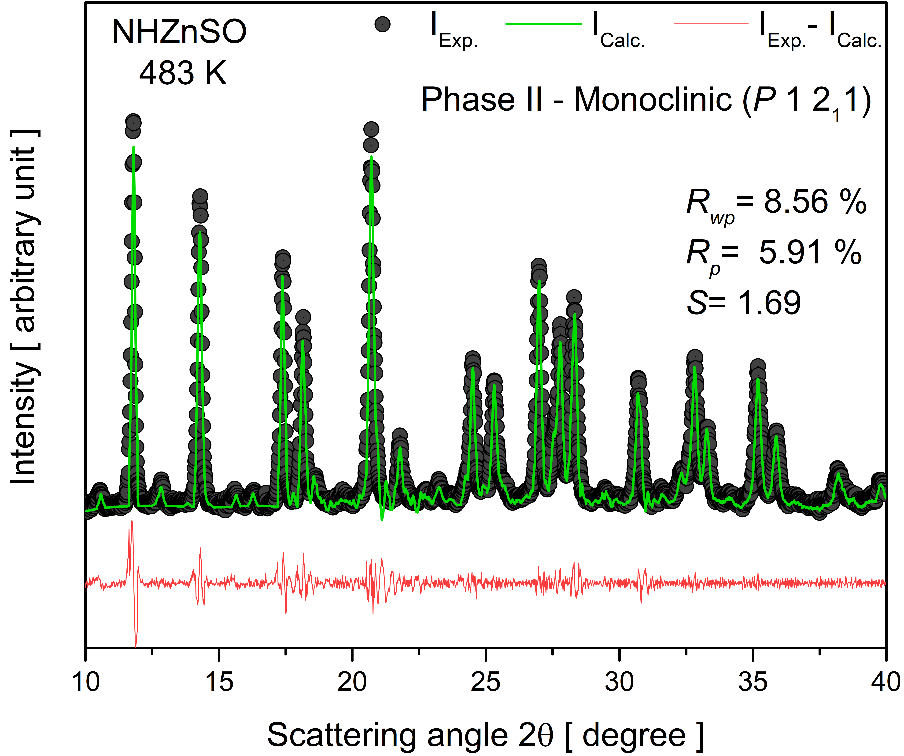
**

**Fig. S3** Refined PXRD pattern by Le Bail method at 483 K.

**Pseudopotential for H atom from file SM.VI__00PBE_OP.recpot**

**A complete description and file SM.VI__00PBE_OP.recpot are available from** <https://www.ccpnc.ac.uk/pspot-site/index.html>

START COMMENT

300 COARSE

500 MEDIUM

650 FINE

PBE OPIUM Pseudopotential H_op_01 generated at Ming-Hsien Lee's Group

=====================================================================

Convergence test for H2

Ecut Energy Energy Diff Bond Bond %

1500 -3.17233675E+001 0.00000000 0.75021321 0.00000000

1400 -3.17228194E+001 5.481000e-004 0.75023979 0.00354280

1300 -3.17223037E+001 0.00106380 0.75025702 0.00584029

1200 -3.17219342E+001 0.00143330 0.75026535 0.00695081

1100 -3.17217700E+001 0.00159750 0.75027063 0.00765367

1000 -3.17216965E+001 0.00167100 0.75028282 0.00927930

900 -3.17211554E+001 0.00221210 0.75032932 0.01547709

800 -3.17186089E+001 0.00475860 0.75051256 0.03990249

700 -3.17102897E+001 0.01307780 0.75114435 0.12411747

600 -3.16875350E+001 0.03583250 0.75287311 0.35455338

500 -3.16312139E+001 0.09215360 0.75643801 0.82973795

400 -3.15029075E+001 0.22046000 0.76143016 1.49516877

300 -3.12289668E+001 0.49440070 0.76416597 1.85983993

Bond length test of molecular H2

===========================================================

| H2 | this pot. (err) | OTFG pot. (err) | Expt. |

-----------------------------------------------------------

| length| 0.750 (1.3%) | 0.751 (1.3%) | 0.7414 |

===========================================================

Bond length test of molecular H2O

===========================================================

| H2O | this pot. (err) | OTFG pot. (err) | Expt. |

-----------------------------------------------------------

| length| 0.967 (1.1%) | 0.972 (1.7%) | 0.956 |

===========================================================

Lattice parameters test of crystalline PdH

===========================================================

| PdH | this pot. (err) | OTFG pot. (err) | Expt. |

-----------------------------------------------------------

| a=b=c | 2.926 (2.9%) | 2.919 (2.7%) | 2.843 |

===========================================================

############################################################

# Opium Parameter File #

############################################################

[Atom]

H

1

100 1.00 -

[Pseudo]

1 1.0

opt

[Optinfo]

7.5 4

[Configs]

3

100 0.75 -

100 0.50 -

100 0.35 -

[xc]

PBE

END COMMENT

**Pseudopotential for N atom from file SM.V_N_00PBE_OP.recpot**

**A complete description and file SM.V_N_00PBE_OP.recpot are available from** <https://www.ccpnc.ac.uk/pspot-site/index.html>

START COMMENT

400 COARSE

500 MEDIUM

700 FINE

PBE OPIUM Pseudopotential N_op_03 generated at Ming-Hsien Lee's Group

=====================================================================

Convergence test for N2

Ecut Energy Energy Diff Bond Bond %

1500 -5.38967429E+002 0.00000000 1.09461630 0.00000000

1400 -5.38967104E+002 3.250000e-004 1.09466222 0.00419475

1300 -5.38966374E+002 0.00105500 1.09465994 0.00398701

1200 -5.38965384E+002 0.00204500 1.09469687 0.00736090

1100 -5.38964760E+002 0.00266900 1.09473848 0.01116203

1000 -5.38964334E+002 0.00309500 1.09475205 0.01240186

900 -5.38962381E+002 0.00504800 1.09474910 0.01213186

800 -5.38958586E+002 0.00884300 1.09483667 0.02013209

700 -5.38954020E+002 0.01340900 1.09482196 0.01878829

600 -5.38910222E+002 0.05720700 1.09534519 0.06658883

500 -5.38517092E+002 0.45033700 1.09970578 0.46495596

400 -5.36505555E+002 2.46187400 1.11881223 2.21044838

300 -5.29540041E+002 9.42738800 1.15047005 5.10258687

Lattice parameters test of crystalline BN

===========================================================

| BN | this pot. (err) | OTFG pot. (err) | Expt. |

-----------------------------------------------------------

| a=b=c | 2.566 (0.4%) | 2.561 (0.2%) | 2.556 |

===========================================================

Lattice parameters test of crystalline TiN

===========================================================

| TiN | this pot. (err) | OTFG pot. (err) | Expt. |

-----------------------------------------------------------

| a=b=c | 2.999 (0.1%) | 3.003 (0.2%) | 2.996 |

===========================================================

Bond length test of molecular N2

===========================================================

| N2 | this pot. (err) | OTFG pot. (err) | Expt. |

-----------------------------------------------------------

| length| 1.095 (-0.3%) | 1.121 (2.2%) | 1.0977 |

===========================================================

Bond length test of molecular NS

===========================================================

| NS | this pot. (err) | OTFG pot. (err) | Expt. |

-----------------------------------------------------------

| length| 1.506 (0.8%) | 1.499 (0.3%) | 1.494 |

===========================================================

Bond length test of molecular NO

===========================================================

| NO | this pot. (err) | OTFG pot. (err) | Expt. |

-----------------------------------------------------------

| length| 1.157 (0.5%) | 1.167 (1.4%) | 1.151 |

===========================================================

############################################################

# Opium Parameter File #

############################################################

[Atom]

N

4

100 2.00 -

200 2.00 -

210 3.00 -

320 -0.01 0.01

[Pseudo]

3 1.45 1.55 1.20

opt

[Optinfo]

7.07 10

7.07 10

7.07 10

[KBdesign]

p

[Configs]

3

#

200 2.00 -

210 3.00 -

320 -0.01 0.01

#

200 1.00 -

210 3.00 -

320 0.5 -

#

200 2.00 -

210 2.00 -

320 0.50 -

#[Pcc]

#0.3

#lfc

[XC]

pbe

[Loginfo]

0

1.6 -4.0 4.0

END COMMENT

**Pseudopotential for O atom from file SM.IV_O_00PBE_OP.recpot**

**A complete description and file SM.IV_O_00PBE_OP.recpot are available from** <https://www.ccpnc.ac.uk/pspot-site/index.html>

START COMMENT

500 COARSE

600 MEDIUM

750 FINE

Pseudopotential generated by OPIUM (Keith Refson)

############################################################

Convergence test for O2

Ecut Energy Energy Diff Bond Bond %

1500 -8.57148384E+002 0.00000000 1.18272864 0.00000000

1400 -8.57147621E+002 7.630000e-004 1.18273398 4.515560e-004

1300 -8.57146017E+002 0.00236700 1.18270064 -0.00236694

1200 -8.57144377E+002 0.00400700 1.18271225 -0.00138533

1100 -8.57143792E+002 0.00459200 1.18277038 0.00352931

1000 -8.57143186E+002 0.00519800 1.18276152 0.00278036

900 -8.57140486E+002 0.00789800 1.18278295 0.00459228

800 -8.57136634E+002 0.01175000 1.18282650 0.00827435

700 -8.57131268E+002 0.01711600 1.18290812 0.01517522

600 -8.56997026E+002 0.15135800 1.18235542 -0.03155527

500 -8.55979302E+002 1.16908200 1.18492723 0.18589187

400 -8.51342496E+002 5.80588800 1.22805192 3.83209444

300 -8.37205383E+002 19.94300100 1.35688591 14.72504051

Lattice parameters test of crystalline CaO

===========================================================

| CaO | this pot. (err) | OTFG pot. (err) | Expt. |

-----------------------------------------------------------

| a=b=c | 3.337 (-1.9%) | 3.336 (-1.9%) | 3.402 |

===========================================================

Lattice parameters test of crystalline Cu2O

===========================================================

| Cu2O | this pot. (err) | OTFG pot. (err) | Expt. |

-----------------------------------------------------------

| a=b=c | 4.268 (-0.1%) | 4.175 (-2.2%) | 4.270 |

===========================================================

Lattice parameters test of crystalline TiO2

===========================================================

| TiO2 | this pot. (err) | OTFG pot. (err) | Expt. |

-----------------------------------------------------------

| a=b | 4.618 (0.7%) | 4.653 (1.4%) | 4.587 |

| c | 2.956 (0.1%) | 2.967 (0.4%) | 2.954 |

===========================================================

Lattice parameters test of crystalline ZrO2

===========================================================

| Zr | this pot. (err) | OTFG pot. (err) | Expt. |

-----------------------------------------------------------

| a=b=c | 3.626 (1.1%) | 3.615 (0.8%) | 3.585 |

===========================================================

Lattice parameters test of crystalline MgO

===========================================================

| MgO | this pot. (err) | OTFG pot. (err) | Expt. |

-----------------------------------------------------------

| a=b=c | 4.272 (1.4%) | 4.159 (-0.2%) | 4.2112 |

===========================================================

Lattice parameters test of crystalline BaTiO3

==========================================================

| BaTiO3| this pot. (err) | OTFG pot. (err) | Expt. |

---------------------------------------------------------

| a=b=c | 4.007 (0.0%) | 4.029 (0.5%) | 4.01 |

==========================================================

Bond length test of molecular CO

===========================================================

| CO | this pot. (err) | OTFG pot. (err) | Expt. |

-----------------------------------------------------------

| length| 1.126 (-0.2%) | 1.142 (1.2%) | 1.128 |

===========================================================

Bond length test of molecular NO

===========================================================

| NO | this pot. (err) | OTFG pot. (err) | Expt. |

-----------------------------------------------------------

| length| 1.157 (0.5%) | 1.167 (1.4%) | 1.151 |

===========================================================

Bond length test of molecular O2

===========================================================

| O2 | this pot. (err) | OTFG pot. (err) | Expt. |

-----------------------------------------------------------

| length| 1.183 (-2.8%) | 1.222 (0.4%) | 1.217 |

===========================================================

Bond length test of molecular H2O

===========================================================

| H2O | this pot. (err) | OTFG pot. (err) | Expt. |

-----------------------------------------------------------

| length| 0.967 (1.1%) | 0.972 (1.7%) | 0.956 |

===========================================================

Lattice parameters test of crystalline Ag2O

===========================================================

| Ag2O | this pot. (err) | OTFG pot. (err) | Expt. |

-----------------------------------------------------------

| a=b=c | 4.777 (1.2%) | 4.861 (3.0%) | 4.720 |

===========================================================

Lattice parameters test of crystalline Ag2O3

===========================================================

| Ag2O3 | this pot. (err) | OTFG pot. (err) | Expt. |

-----------------------------------------------------------

| a=b=c | 5.054 (2.5%) | 5.135 (4.2%) | 4.930 |

===========================================================

############################################################

# Opium Parameter File #

############################################################

[Atom]

O

3

100 2.00 -

200 2.00 -

210 4.00 -

[Pseudo]

2 1.34 1.53

opt

[Optinfo]

7.07 10

7.07 10

conmax

[Configs]

2

#

200 2.00 -

210 4.20 -

#

200 2.00 -

210 4.40 -

[XC]

gga

END COMMENT

**Pseudopotential for S atom from file SM.III_S_00PBE_OP.recpot**

**A complete description and file SM.III_S_00PBE_OP.recpot are available from** <https://www.ccpnc.ac.uk/pspot-site/index.html>

START COMMENT

300 COARSE

400 MEDIUM

650 FINE

PBE OPIUM Pseudopotential S_op_02 generated at Ming-Hsien Lee's Group

=====================================================================

Convergence test for S2

Ecut Energy Energy Diff Bond Bond %

1500 -5.53839266E+002 0.00000000 1.91433739 0.00000000

1400 -5.53837446E+002 0.00182000 1.91425327 -0.00439409

1300 -5.53834008E+002 0.00525800 1.91423828 -0.00517733

1200 -5.53828613E+002 0.01065300 1.91427410 -0.00330606

1100 -5.53821429E+002 0.01783700 1.91454430 0.01080853

1000 -5.53813737E+002 0.02552900 1.91464924 0.01629017

900 -5.53807335E+002 0.03193100 1.91464475 0.01605587

800 -5.53802093E+002 0.03717300 1.91503875 0.03663728

700 -5.53793571E+002 0.04569500 1.91598802 0.08622483

600 -5.53776289E+002 0.06297700 1.91731221 0.15539675

500 -5.53743584E+002 0.09568200 1.92003574 0.29766701

400 -5.53631400E+002 0.20786600 1.92377278 0.49288015

300 -5.53012902E+002 0.82636400 1.92801622 0.71454681

Lattice parameters test of crystalline FeS2

===========================================================

| FeS2 | this pot. (err) | OTFG pot. (err) | Expt. |

-----------------------------------------------------------

| a=b=c | 5.351 (-1.4%) | 5.365 (-1.2%) | 5.428 |

===========================================================

Lattice parameters test of crystalline GeS2

===========================================================

| GeS2 | this pot. (err) | OTFG pot. (err) | Expt. |

-----------------------------------------------------------

| a=b=c | 6.139 (2.4%) | 6.118 (2.1%) | 5.993 |

===========================================================

Lattice parameters test of crystalline PtS

===========================================================

| PtS | this pot. (err) | OTFG pot. (err) | Expt. |

-----------------------------------------------------------

| a=b | 3.542 (1.8%) | 3.551 (2.0%) | 3.480 |

| c | 6.157 (0.7%) | 6.150 (0.7%) | 6.110 |

===========================================================

Bond length test of molecular S2

===========================================================

| S2 | this pot. (err) | OTFG pot. (err) | Expt. |

-----------------------------------------------------------

| length| 1.914 (1.3%) | 1.899 (0.5%) | 1.8892 |

===========================================================

Bond length test of molecular NS

===========================================================

| NS | this pot. (err) | OTFG pot. (err) | Expt. |

-----------------------------------------------------------

| length| 1.506 (0.8%) | 1.499 (0.3%) | 1.494 |

===========================================================

############################################################

# Opium Parameter File #

############################################################

[Atom]

S

6

100 2.00 -

200 2.00 -

210 6.00 -

300 2.00 -

310 4.00 -

320 -0.01 -0.01

[XC]

pbe

#[Pcc]

#0.00

#lfc

[Pseudo]

3 1.6 1.40 1.40

opt

[Optinfo]

5.50 5

6.00 4

7.00 4

[KBdesign]

s

[Configs]

2

#

300 2.00 -

310 3.50 -

320 0.00 -

#

300 1.50 -

310 3.50 -

320 0.00 -

[loginfo]

0

1.65 -4.0 4.0

END COMMENT

**Pseudopotential for Zn atom from file SM.II_Zn_00.recpot**

**A complete description and file SM.II_Zn_00.recpot are available from** <https://www.ccpnc.ac.uk/pspot-site/index.html>

START COMMENT

380 COARSE

460 MEDIUM

600 FINE

Zn002 (Ming-Hsien Lee, Sep 1994)

----------------------------------

Qc-tuned Projector Reduced Optimized Pseudopotential Zn002

Electronic configuration :

For s p and d : 3d 10.00 4s 1.27 4p 0.73

Rc(s,p,d) = (2.0, 2.0, 2.4)

Qc/q3(s,p,d) = (0.78, 0.965, 1.2125)

Local : mixed 0.2*Vs + 0.8*Vp

Projector reduced for s and p

Tests:

Convergence tests for a Zn dimer molecule, box of 7x7x7 A,

LDA, Gamma point sampling. Eunrel refers to the total energy

for the bond length of 2.386 A (OFF result), Erel is the total

energy at the equilibrium geometry, and Ecut is the plane wave

cutoff energy.

Ecut Eunrel Erel Bond

300 -2533.89880 -2534.21020 2.928

350 -2559.96107 -2560.29362 3.354

400 -2563.55340 -2563.87427 3.095

450 -2563.80518 -2564.12621 3.076

500 -2563.85632 -2564.17578 3.297

600 -2564.06871 -2564.38831 3.233

700 -2564.08555 -2564.40506 3.206

END COMMENT
